# Molecular Mechanism of Nucleosome Recognition by the Pioneer Transcription Factor Sox

**DOI:** 10.1021/acs.jcim.2c01520

**Published:** 2023-06-12

**Authors:** Burcu Ozden, Ramachandran Boopathi, Ayşe Berçin Barlas, Imtiaz N. Lone, Jan Bednar, Carlo Petosa, Seyit Kale, Ali Hamiche, Dimitar Angelov, Stefan Dimitrov, Ezgi Karaca

**Affiliations:** † Izmir Biomedicine and Genome Center, Dokuz Eylul University Health Campus, Izmir 35340, Turkey; ‡ Izmir International Biomedicine and Genome Institute, Dokuz Eylül University, Izmir 35340, Turkey; § Institut for Advanced Biosciences, Inserm U 1209, CNRS UMR 5309, Université Grenoble Alpes, Grenoble 38000, France; ∥ Institut de Biologie Structurale (IBS), Université Grenoble Alpes, CEA, CNRS, Grenoble 38044, France; ⊥ Laboratoire de Biologie et de Modélisation de la Cellule (LBMC), Université de Lyon, Ecole Normale Supérieure de Lyon, CNRS, 46 Allée d’Italie, Lyon 69007, France; # Département de Génomique Fonctionnelle et Cancer, Institut de Génétique et Biologie Moléculaire et Cellulaire (IGBMC)/Université de Strasbourg/CNRS/INSERM, Illkirch Cedex 67404, France; ∇ Roumen Tsanev Institute of Molecular Biology, Bulgarian Academy of Sciences, 1113 Sofia, Bulgaria

## Abstract

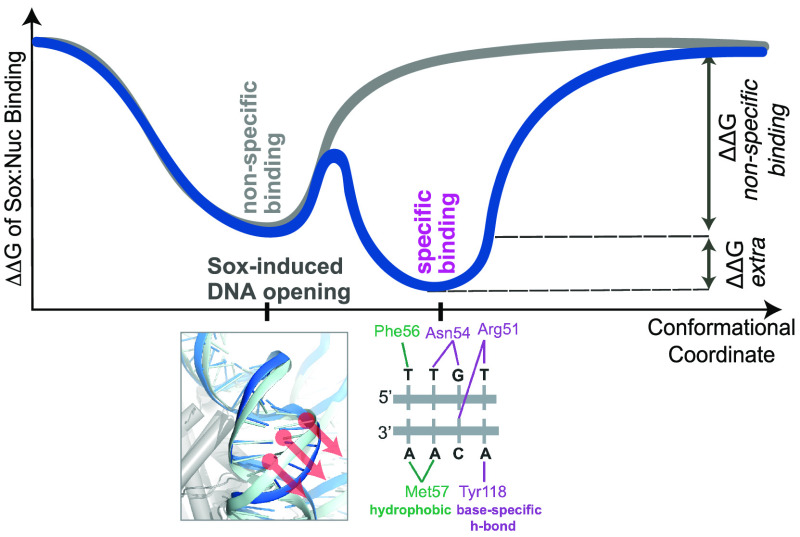

Pioneer transcription factors (PTFs) have the remarkable ability to directly bind to chromatin to stimulate vital cellular processes. In this work, we dissect the universal binding mode of Sox PTF by combining extensive molecular simulations and physiochemistry approaches, along with DNA footprinting techniques. As a result, we show that when Sox consensus DNA is located at the solvent-facing DNA strand, Sox binds to the compact nucleosome without imposing any significant conformational changes. We also reveal that the base-specific Sox:DNA interactions (base reading) and Sox-induced DNA changes (shape reading) are concurrently required for sequence-specific nucleosomal DNA recognition. Among three different nucleosome positions located on the positive DNA arm, a sequence-specific reading mechanism is solely satisfied at the superhelical location 2 (SHL2). While SHL2 acts transparently for solvent-facing Sox binding, among the other two positions, SHL4 permits only shape reading. The final position, SHL0 (dyad), on the other hand, allows no reading mechanism. These findings demonstrate that Sox-based nucleosome recognition is essentially guided by intrinsic nucleosome properties, permitting varying degrees of DNA recognition.

## Introduction

The nucleosome core particle (NCP) is the basic repeating unit of the eukaryotic genome.^1^ The NCPs, connected with the linker DNA, make up the 10 nm chromatin filament, which folds into higher-order chromatin structures upon binding to the linker histone.^2−5^ The NCP comprises a core histone octamer (twice the H2A, H2B, H3, and H4 proteins) and 147 base pairs (bp) of DNA. The 147 bp DNA wraps around the histone octamer in 1.67 left-handed helical turns,^1^ of which positioning on NCP is described by the superhelical locations (SHLs). The SHLs are counted in the positive and negative directions, starting from the central DNA sequence, i.e., the nucleosomal dyad (SHL0). They are separated by ∼10 bp nucleotides while running from ±7 to ±1 positions.^6^ At each SHL, the accessibility of DNA for proteins is regulated by chromatin remodelers.^7^ The remodelers aid the freeing of nucleosomal DNA from histones to make specific recognition sequences available for transcription factors (TFs).^8,9^ In the absence of remodelers, nucleosomes present an impediment to transcription, as the vast majority of TFs cannot overcome the nucleosomal barrier to recognize their binding sequence.^10−15^ An exception to this rule is the pioneer transcription factors (PTFs). Unlike conventional TFs, PTFs directly bind to nucleosomally organized DNA to assist the assembly of complex transcriptional machineries.^16−18^ This fact delegates PTFs a central role in essential chromatin-templated processes, such as establishing competence for gene expression and initiating cellular programming.^19^

**Figure 1:**
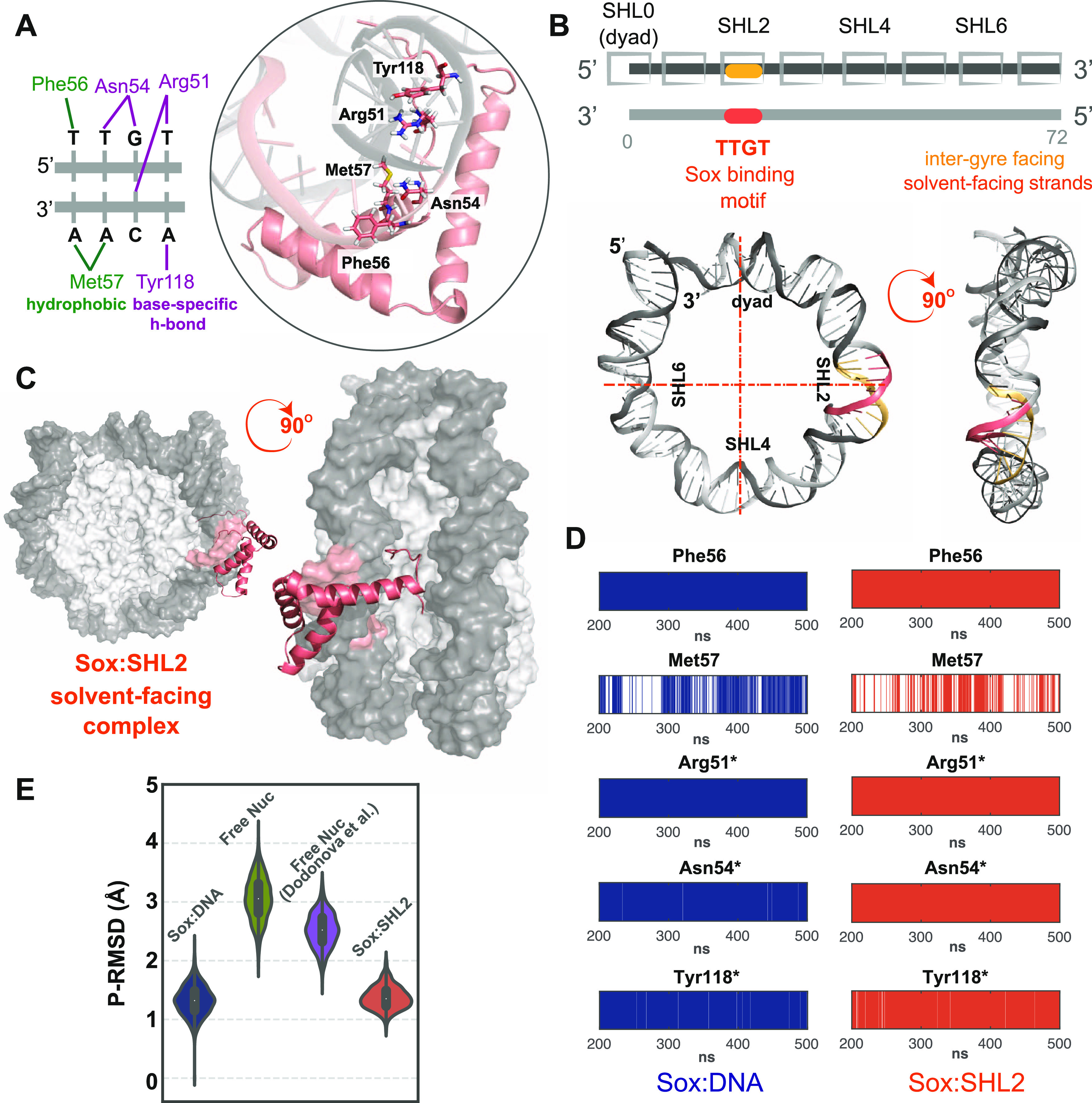
(A) Important Sox:DNA interactions. The Sox:DNA interactions reported to be critical to Sox:DNA recognition are demonstrated in sticks on Sox11:DNA complex (PDB id: 6T78). The core 5′-TTGT-3′ and its complementary Sox binding motif are specifically recognized by hydrogen bonds formed by Arg51, Asn54, and Tyr118 and hydrophobic interactions by Phe56 and Met57. Hydrophobic interactions and base-specific hydrogen bonds are colored in green and purple, respectively. (B) Sox cognate sequence can be placed either on the solvent-facing or intergyre-facing strand. The representative coordinates of each positive SHL are highlighted with a box. The corresponding solvent-facing (salmon) or intergyre-facing (orange) Sox cognate sequence (5′-TTGT-3′) positioning at SHL2 is shown on the nucleosomal DNA. (C) Solvent-facing Sox11:SHL2 complex (front and side views). Histones and nucleosomal DNA are represented in surface and colored in light and dark gray, respectively. Sox is depicted as cartoon and colored in salmon. (D) Sox11:DNA (dark blue) and Sox11:SHL2 (salmon) interaction profiles of the essential Sox amino acids. Each barcode plot shows the presence of the denoted amino acid interaction with the DNA (within the equilibrated simulation time frame). The prevalent base-specific interactions formed by Arg51, Asn54, and Tyr118 are presented for Sox11:DNA and Sox11:SHL2, respectively, and highlighted with an asterisk. (E) Molecular dynamics (MD)-driven phosphate root-mean-square deviations (P-RMSDs) from the native Sox-bound-DNA conformation (PDB id 6T78). The P-RMSD distributions of the Sox cognate sequence, derived from Sox11:DNA (dark blue, *N* = 1200), free nucleosomal SHL2 601 DNA (green, *N* = 3000), free nucleosomal SHL2 NCAP-SELEX DNA (purple, *N* = 1200), and Sox11:SHL2 model (salmon, *N* = 1200) simulations. The persistence rate of each interaction is provided in Table S1.

During the past decade, several studies explored the interaction landscape between PTFs and NCP.^16−18,20^ Accordingly, PTFs are proposed to have a high mobility group (HMG), forkhead, Pit-Oct-Unc (POU), zinc finger (ZF), and basic helix–loop–helix (bHLH) DNA-binding domains.^21,22^ Among these, the HMG domain is composed of 79 amino acids. It folds into a simple three-helix architecture arranged in a boomerang L-shape, residing between 10 residues long N- and C-terminal tails^23−26^ (Figures 1A and S1). Like the other PTFs, HMGs are demonstrated to guide a diverse range of vital processes.^27−32^ For example, the HMG domain carrying Sox2 was shown to be a part of the TF cocktail, capable of inducing pluripotent stem cells from somatic human cells.^33,34^ Another HMG protein, Sox4, is a crucial factor in the epithelial-to-mesenchymal transition, a fundamental process operating in cancer progression and metastasis.^35,36^ Together with the other 18 proteins, Sox2/4 make up the Sox family, the so-called “ultimate utility player of the cell”.^33,37^ For the sake of simplicity, from this point and on, we will refer to Sox-HMG as Sox. The Sox proteins recognize their cognate 5′-TTGT-3′ sequence, positioned at a DNA minor groove, through the base and shape reading mechanisms, i.e., they form base-specific hydrogen bonds with 5′-TTGT-3′ while imposing an extreme DNA distortion induced by two hydrophobic residues of Sox^37−43^ (Figure 1A). The bent DNA is further stabilized through ionic interactions, established between the basic residues of N- and C-terminal tails of Sox and the DNA backbone (Figure S1). This base and shape DNA reading mechanisms of Sox is invariant among all Sox:DNA complexes.^44^

In 2020, Dodonova et al. unveiled the nucleosome recognition mode of Sox2 and Sox11 at the atomistic detail.^45^ In their experiments, they used a nucleosomal DNA obtained by the NCAP-SELEX method.^20^ This DNA sequence harbored Sox-binding-enriching patterns, where the cognate 5′-TTGT-3′ was located at several solvent-exposed intergyre positions (Figure 1B). Among different SHL sites, Sox2 and Sox11 could bind only to SHL2 (PDB ids: 6T7A and 6T7B). In both Sox:nucleosome complexes, the binding of Sox was accompanied by a strong local distortion of the nucleosomal DNA, involving a 7 Å widening of the minor groove. The tight interactions between Sox and nucleosomal DNA resulted in the pulling of DNA away from the histone octamer by ∼4 Å. In this way, Sox could bind at SHL2 in the same way as in free DNA. As an intriguing observation, in both structures, the binding-induced detachment of 25 bp double-stranded (ds) DNA end (at SHL-5, SHL-6, and SHL-7 positions) was observed due to the steric clashes formed between Sox11 and the adjacent gyre (Figure S2A,B). At higher Sox11 concentrations, a second Sox11 could specifically bind to NCP, but this time, at SHL2. This binding resulted in the freeing of an additional 25 bp dsDNA from the other nucleosomal DNA end (at SHL5, SHL6, and SHL7). The binding of Sox11 also was associated with a displacement of the N-terminal H4 tail as Sox C-terminal tail’s conformation was not compatible with it. By utilizing these structural displacements, Sox was proposed to alter the canonical nucleosome–nucleosome contacts to locally open the chromatin fiber.^45^

In 2020, Michael et al. resolved another mode of Sox2 interaction with NCP, this time in the presence of Oct4.^46^ In their experiments, they used coupled Oct4–Sox2 binding motifs at several intergyre-facing SHLs within 601 nucleosomal DNA. The 601 nucleosome has a strong positioning DNA sequence, which ensures having the same histone–DNA registry in every experiment.^47^ The 601 DNA has a nonpalindromic sequence, where the two DNA halves share 28% sequence similarity. As a result of their experiments, Michael et al. could detect a significant Sox2 binding only in cooperation with Oct4 at SHL±6 sites. At the SHL-6 site, Sox binding motif was placed toward the entry/exit site of the nucleosome, while at the SHL6 site, the nucleosomal DNA ended with Oct4 binding sequence. Their high-resolution cryoelectron microscopy (cryo-EM) structure showed that at the left DNA end (SHL-6), the binding of Sox2–Oct4 led to the release of DNA from the histone octamer, while binding at the other end (SHL6) resulted in local DNA distortions. These results highlight the impact of the order in motif placement (Sox2–Oct4 or Oct4–Sox2) in determining the differential perturbation effects on nucleosomal DNA.

Expanding on these structural investigations, in 2022, Malaga Gadea and Nikolova utilized NMR and other biophysical experiments to unravel the nucleosome recognition mechanism of Sox2.^48^ Like in Michael et al.’s experiments, they made use of a modified 601 nucleosome. In this case, they systematically inserted Sox2 and Oct4 binding sequences in different orders on the positive nucleosomal DNA arm. As a result, they revealed that Sox2 binding is strongly position-dependent, where the most stable (high affinity) Sox binding was detected at the intergyre-facing SHL5 motif. Here, high-affinity SHL5 binding was characterized by two aspects, large DNA bending and stable Sox tail–histone interactions. A weaker Sox binding was also observed at SHL2 and SHL6. At other positions, nonspecific Sox binding was observed as well. In this work too, only cooperative Sox2 and Oct4 interaction led to a coupled Sox2–Oct4 binding at the DNA entry–exit sites. Finally, Malaga Gadea and Nikolova did not detect extreme DNA end detachment in their experiments.

In parallel to these experimental efforts, a handful of computational approaches investigated how PTFs could recognize their cognate sequence on a compact nucleosome. Within this context, Huertas et al. investigated how sequence-dependent nucleosomal DNA dynamics could impact PTF binding.^49,50^ For this, they carried out several 1 μs long atomistic molecular dynamics (MD) simulations of 601 NCP and two natural nucleosomes, ESRRB and LIN28B, in the presence and absence of histone tails. ESRRB contains one Oct4 binding site and the same AT content as the 601 nucleosome (45.2% vs 42.3%). LIN28B has a higher AT content (59.5%) and multiple Oct4 and other TF binding sites. The exploration of comparative dynamics of these nucleosomes revealed that (1) the DNA flexibility of these nucleosomes follows LIN28B > 601 > ESRRB, (2) the overall nucleosome flexibility is similar in complete and tail-less nucleosomes. These outcomes elucidated that the nucleosome dynamics is predominantly impacted by the thermal fluctuation range of nucleosomal DNA. They also showed that a natural sequence such as ESRRB could be less flexible compared to the strong positioning 601 sequence. Following the exploration of their comparative nucleosomal DNA dynamics, Huertas et al. modeled Oct4 binding on a LIN28B nucleosome, where only certain Oct4 orientations could fit the structural restrictions posed by the nucleosomes.

In another computational study, carried out by Tan and Takada,^51^ coarse-grained MD was used to dissect the binding mechanism of Sox2 and Oct4 to 601 and LIN28B nucleosomes. By having the 5′-TTGT-3′ sequence integrated at multiple SHL sites, they found that Sox2 can stably bind to its intergyre-facing sequence in 601, placed at SHL3. They also observed off-target binding at various locations, including the dyad. Tan and Takada also claim that the specific recognition of Sox cognate sequence is directly correlated with the bendability of DNA, which is coupled with the local disruption of histone–DNA contacts. For the SHL3 binding, they did not observe any large-scale DNA end detachments. In the case of the LIN28B nucleosome, Sox2 could not stably bind to its cognate sequence as its binding motif was only partially solvent exposed. Interestingly, Tan and Takada could not detect a position dependency for Oct4. They also discussed that Sox binding induces allosteric effects for Oct4 recognition, as observed in the other studies.

Thanks to the above-described experimental and computational efforts, we now know that the mononucleosome invasion capacity of Sox depends on two factors, i.e., the location of Sox binding motif and the nucleosomal DNA dynamics (Table 1). Though, to completely grasp the universal binding rules of Sox, the following fundamental question should be answered: Can Sox read its binding sequence, even when it is placed on the DNA strand complementary to what has been already probed (i.e., the intergyre-facing strand, Table 1)? To explore this fundamental question, we designed a dynamic integrative modeling (DIM) approach focused on the detailed investigation of nucleosomal DNA dynamics at different resolutions. For this, we combined in silico (integrative modeling and classical MD simulations) and experimental (^•^OH and UV laser DNA footprinting) techniques. With these tools, we initially explored whether the solvent-facing Sox binding at SHL2 could be realized in a sequence-specific manner, as in Dodonova et al.^45^ We then researched whether solvent-facing motif placement would allow Sox binding at two other locations on the positive DNA arm, i.e., SHL4 and SHL0 (dyad), for which specific Sox binding has not been recorded.

**Table 1:** Described Sox:Nucleosome Binding Patterns

reference	approach	nuc. DNA sequence	motif placement	observed binding site	Sox type	outcome
Dodonova et al.^45^	cryo-EM	NCAP-SELEX	intergyre	SHL±2	Sox2, Sox11	25 bp DNA end detachment
Michael et al.^46^	cryo-EM	modified 601	intergyre	SHL±6	Sox2, Oct4	DNA end detachment when Sox binds toward entry/exit
Malaga Gadea and Nikolova^48^	NMR, cross-linking, EMSA, footprinting	modified 601	intergyre	SHL5 (strong), SHL2 (weak), SHL6 (weak)	Sox2	local DNA distortions
						nonspecific binding
Tan and Takada^51^	coarse-grained MD	modified 601	intergyre	SHL3	Sox2	local DNA distortions
						nonspecific binding

## Results

### MD Simulations of Sox:DNA Complex Reveals the Essential Protein–DNA Interaction Dynamics

The earliest Sox:DNA complex dates back to 1995.^43^ Since then, a number of other Sox:DNA complexes have been characterized. These complexes showed that Sox binds to the minor groove, while inducing a dramatic deformation at its recognition sequence, 5′-TTGT-3′ (Figure 1A). In all of these complexes, the regions inducing these changes were defined as the hydrophobic FM wedge (Phe56, Met57) and the polar Asn54 (numbering follows the one in 6T78 PDB).^39−43,45^ Among these amino acids, Phe56 triggers the minor groove opening, while the 60–70° bending is maintained by the base-specific Asn54:DNA interactions with the central 5′-TG-3′ motif (Figure 1A). In Sox-bound state, the maximum DNA minor groove opening increases up to 22.5 Å. Next to these shape-preserving essential contacts, the core 5′-TTGT-3′ Sox sequence is further read by Arg51 and Tyr118. All of the critical amino acids except for one are positioned at the core of Sox, while the exception, Tyr118, is located on the C-terminal tail of Sox. These three residues form base-specific hydrogen bonds with the target and the complementary binding motif, as presented in Figure 1A. To understand the specificity and persistence of these interactions, we performed (two replicas of 500 ns long) MD simulations of the Sox11:DNA complex (PDB id: 6T78).^45^

As a result, we observed that Arg51, Asn54, and Tyr118 interact with 5′-TTGT-3′ during the whole simulation time in a base-specific manner (Figure 1D—blue profiles and Table S1). Furthermore, we saw that, from the FM wedge, Phe56 is the one making persistent hydrophobic interactions, while Met57 contacts DNA during 50% of the simulation time (Figure 1D—blue profiles and Table S1). This analysis places Phe56, Arg51, Asn54, and Tyr118 as indispensable to specific Sox11:DNA recognition. We also noted that the Sox-bound-DNA backbone conformation fluctuates around its initially crystallized state within 0.7–2.2 Å phosphorus root-mean-square deviation (P-RMSD (Methods section, Figure 1E—blue distribution)), defining the DNA thermal fluctuation range permitted by Sox binding. We expect that any native Sox:nucleosome complex should reflect the same base-specific contact (base reading) and DNA thermal fluctuation (shape reading) profile.

### Sox Can Exert Its Fingerprint Base and Shape DNA Reading at SHL2

To probe the universal nucleosome recognition mechanism of Sox, we structurally modeled a new nucleosome upon concurrently inserting the core 5′-TTGT-3′ Sox binding sequence at dyad, SHL2, and SHL4 sites of the 601 nucleosome (PDB id: 3LZ0(47)). Here, we placed an SHL gap between the Sox binding sequences to ensure that these newly incorporated sites will be distant enough not to “feel” each other. At each mutated SHL site, the core Sox-binding sequence was inserted on the solvent-facing nucleosomal DNA strand (Figures 1B and S2A,B). Our motivation in constructing a derivate of the stable 601 nucleosomal DNA sequence was, first, to have a well-behaving system for experimental validation, and second, to focus only on the conformational freedom injected into the system upon incorporating Sox-binding sites. In its initial conformation, neither the mutated 601 nor any available free nucleosome structure could accommodate solvent-facing Sox binding due to the natural narrow geometry of their minor grooves (Figure S2C, D). To explore the thermal fluctuation range of mutated 601 beyond the known conformational space of nucleosomal DNA, we performed two 1 μs long MD simulations of the mutant 601. During our simulations, we did not explicitly model histone tails to save sampling time and to alleviate potential force field problems that could occur due to the suboptimal tail–DNA interaction modeling.^52−54^ This choice is also backed up by the literature information on comparative nucleosomal DNA dynamics explored by Huertas et al., as highlighted in the Introduction section.^50^

As an outcome of our mutated 601 simulations, we saw that Sox-binding sites very rarely open wide enough to match the 0.7–2.2 Å Sox-bound-DNA P-RMSD range (SHL2 site in Figure 1E—blue profile, SHL0/2/4 sites in Figure S2F). Furthermore, 22.5 Å minor groove widening was never observed across the nucleosome positions (Figure S3A). To serve as a reference, we simulated the free 6T79 NCAP-SELEX nucleosome, the nucleosomal DNA sequence of the intergyre-facing Sox:nucleosome complex^20^ (two 500 ns long MD simulations). The comparative analysis of our mutated 601 and 6T79 simulations revealed that (1) both complexes reflect similar global RMSDs from their initial states (Figure S3A), (2) in both nucleosomes, at the SHL2 site, DNA minor groove widening and lowest P-RMSD values point to a too-narrow site for Sox binding (Figures 1E—green and purple distributions and S3A), (3) the position-specific DNA fluctuation ranges are similar (Figure S3B). These similarities underscored the validity of using our mutant nucleosome for further modeling.

Since we know that Sox can recognize its binding sequence at SHL2, from our mutated 601 nucleosome simulations, we isolated the nucleosome state fitting best to Sox-bound-DNA at the SHL2 site (with 1.9 Å P-RMSD, Figure S2E). By taking this best-fitting SHL2-nucleosome and the bound conformation of Sox11 (from 6T78), we imposed the known Sox11:DNA interactions in HADDOCK and obtained an initial Sox:SHL2 model^55,56^ (Figure 1C, Methods section). This model was subjected to two rounds of 500 ns long MD simulations to allow enough time for Sox11 to find its native binding pose. As a result, we observed that the Sox11:SHL2 complex behaves stably during the last 300 ns of the simulations (Figure S4A), which we took as a basis for the rest of our analyses. Tracing the P-RMSD profile of the Sox11:SHL2 complex and comparing it to the 601-SHL2 P-RMSD profile revealed something very striking: Sox binding is indispensable to induce the extreme minor groove geometry (Figures 1E and S4B). So, only when Sox11 is located at its binding site, the P-RMSD distribution of Sox-bound-DNA profiles could be replicated. In this state, the minor groove widths fluctuate around the one of experimental Sox11:nucleosome structure. When we concentrated on the specific interaction profiles of Sox, we observed that at SHL2, the critical amino acids of Sox behave exactly the same as in the case of Sox:DNA complex (Figure 1D and Table S1). This implies that SHL2 acts transparently to Sox binding when Sox cognate motif faces the solvent. This observation told us that the persistent Sox:DNA interaction network directed by Phe56, Arg51, Asn54, and Tyr118 is required to induce the extreme minor groove deformation also on the nucleosome. As obtaining these observations on the Sox11:SHL2 model required the incorporation of integrative structural modeling, as well as running several cycles of MD simulations, we named our protocol after dynamic integrative modeling (DIM, Methods section). At SHL2, our Sox-bound nucleosomal DNA and Dodonova et al.’s Sox-bound nucleosomal DNA structures are of the same distance to Sox:free DNA complex (P-RMSD: 1.4 Å), endorsing the robustness of our approach.

**Figure 2:**
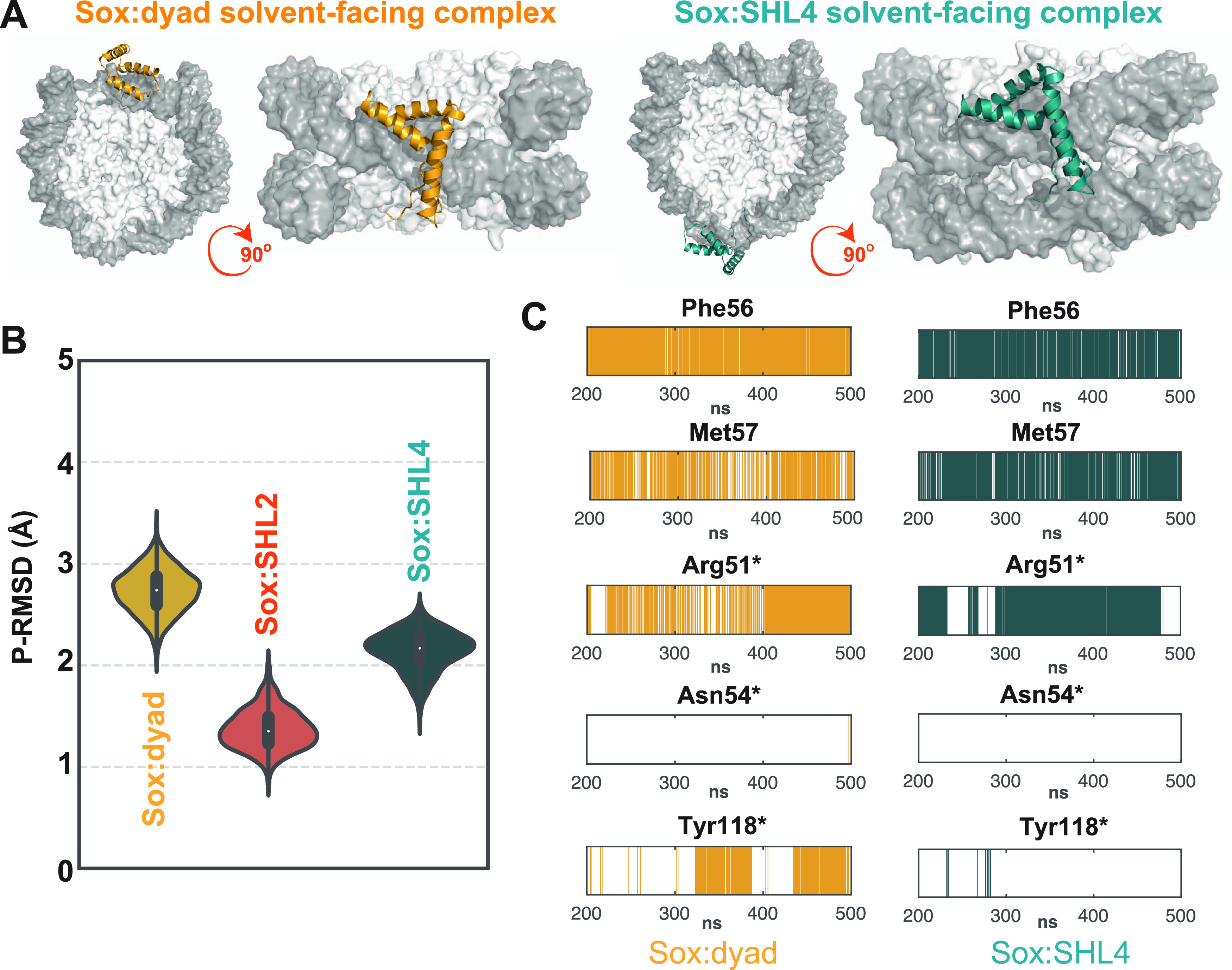
(A) Front and side views of solvent-facing Sox11:dyad (orange) and Sox11:SHL4 (green) complex models. Histones and nucleosomal DNA are represented as surface and colored in light and dark gray, respectively. (B) MD-driven P-RMSDs of each Sox11:SHL complex compared to the Sox-bound-DNA conformation. P-RMSD distributions of the Sox cognate sequence, derived from Sox11:dyad (orange, *N* = 1200), Sox11:SHL2 (salmon, *N* = 1200), and Sox11:SHL4 (dark green, *N* = 1200) complex simulations. The Sox11:SHL2 values are a replicate of Figure 1E and are placed here to serve as a basis for comparison. (C) Sox11:DNA interaction profiles of the essential Sox amino acids at dyad and SHL4 (*N* = 600). Each barcode plot shows the persistence of the denoted amino acid interaction with DNA (within the equilibrated simulation time frame). The prevalent base-specific interactions formed by Arg51, Asn54, and Tyr118 are highlighted with asterisks. The persistence rate of each interaction is given in Table S1.

To reveal the position-dependent dynamic behavior of nucleosomal DNA, we modeled Sox11 binding at SHL4 and dyad (Figure 2A) following the same DIM protocol as described above. Comparing the global RMSD profiles of all Sox11 complexes showed that the highest RMSD fluctuations are visited by Sox11:SHL4 (Figure S4A). This global analysis also revealed that the Sox11:SHL4 complex could barely satisfy the fingerprint minor groove width profile of Sox-DNA (Figure S4B). Accordingly, Sox11:SHL4 reflects Sox-bound-SHL2-like DNA conformations only during 28% of the simulation time (Figure 2B). From the critical interactions point of view, at SHL4, the hydrophobic Met57:DNA interactions are increased by 60% (Figure 2D and Table S1). Strikingly, in this case, the base-specific Asn54 and Tyr118 hydrogen bonds are lost. Moreover, base-specific Arg51 interactions are observed only in one replica simulation (Table S1 and Figure S4C). In the case of Sox:dyad, the simulated conformers could not even reach the fingerprint minor groove peak at 22.5 Å (Figure S4B). Accordingly, Sox11:dyad almost never exerts the Sox-bound-DNA conformation (Figure 2B). We also found that in the case of Sox11:dyad, the specific Asn54 interactions are lost, and the specific Tyr118 interactions are reduced by 65%, while the hydrophobic Met57 interactions are increased by 38% (Figures 2C—left and S4C and Table S1). These findings indicate that the more specific Sox:DNA interactions are lost, the more hydrophobic nonspecific interactions prevail. They also imply that SHL4 permits solely shape reading during ∼30% of simulation time, and dyad allows no reading mechanism at all.

### Solvent-Facing Sox Binding Is Confirmed by the ^•^OH Footprinting Experiments

**Figure 3:**
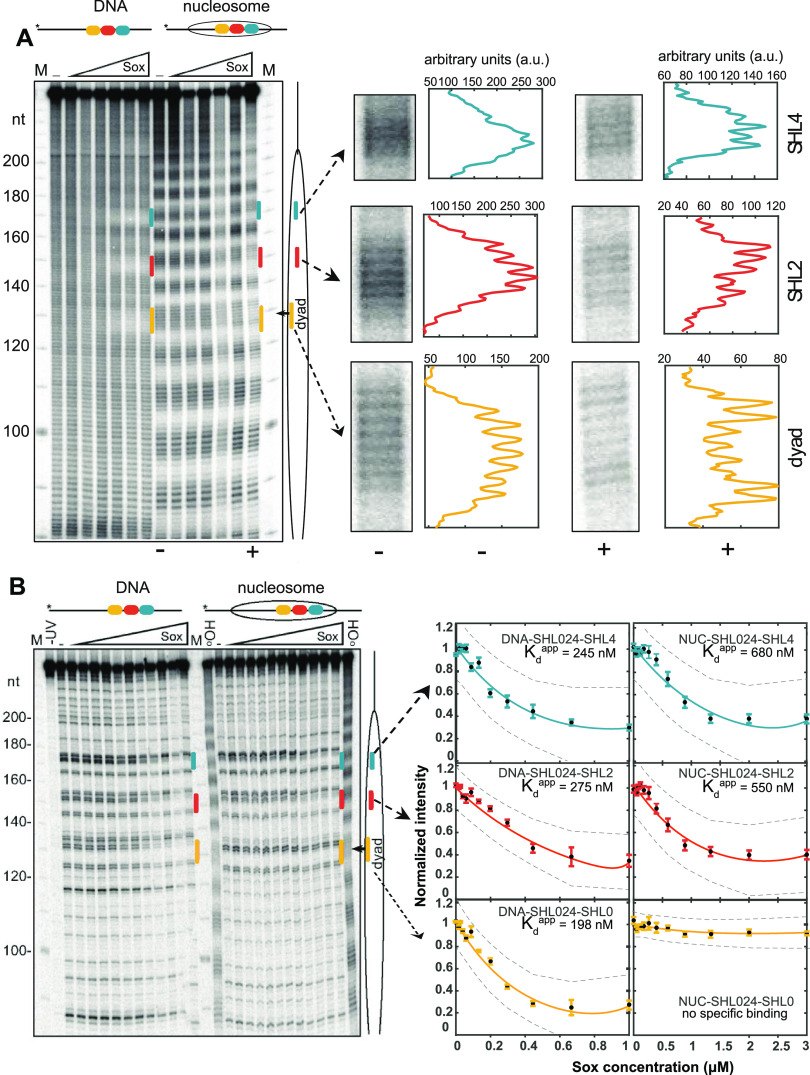
(A) Left panel: ^•^OH radical footprinting patterns of Sox:DNA and Sox:nucleosome complexes bearing the three recognition sites of Sox (601-SHL024). After ^•^OH treatment of the complexes, the cleaved DNA fragments were purified and separated on 8% sequencing gel. They were then visualized by autoradiography. (−, +) sign indicates the presence and absence of Sox6 protein. Right panel: Vertical colored lines correspond to Sox-binding sites, where the oval representes the nucleosome. Higher magnification and quantification of the indicated Sox-binding sites. ^•^OH cleavage patterns of unbound (−) and Sox6-bound particles (+), containing the three Sox recognitions sites. (B) Left panel: UV laser footprinting patterns of Sox6:DNA and Sox6:nucleosome complexes (601-SHL024). The first line shows the ^•^OH cleavage patterns of the unbound particle. The complex was irradiated with a single 5 ns UV laser 266 nm pulse (Epulse, 0.1 J/cm^2^), and DNA was purified from the samples. After treatment of the purified DNA with Fpg glycosylase, the cleaved DNA fragments were separated on 8% sequencing gel and visualized by autoradiography. Red vertical lines and red squares mark the Sox-binding sites, M marks the molecular mass, the oval represents the schematics of the nucleosome, and the dyad is indicated with an arrow. −UV refers to the control, non-UV irradiated, and Fpg-treated sample. Right panel: (left) Sox6 concentration dependencies of the footprinting intensities, representing the normalized cleavage band intensity of the GG run within the binding site; (right) The normalized intensity profiles based on our gel quantifications were plotted as a function of Sox6 concentration. The equation used for the curve fitting was *f*1(*x*) = *a* exp(*b* × *x*) + *c* exp(*d* × *x*). The equation parameters for each curve fitting are provided in the Methods section.

To confirm the solvent-facing Sox binding at the dyad, SHL2, and SHL4, we used hydroxyl radical (^•^OH) footprinting. The ^•^OH footprinting is a versatile technique to analyze the binding of Sox to NCP, as ^•^OH radicals attack DNA via the minor groove.^57−59^ As Sox binds to the DNA minor groove, a footprint should be visible at each relevant SHL site. Expanding on this, we performed ^•^OH footprinting on our mutated 601 sequence, in isolation and when wrapped around the histones (Methods section; Figures 3A and S5). In these experiments, instead of Sox11, we used Sox6 to trace Sox binding, as it stably behaved during our experiments. Even though Sox6 does not have any experimentally determined structure, we permitted this as Sox binding to DNA is strictly conserved across the Sox family. This is also reflected by the identity and similarity percentages of HMG domains shared between Sox11 and Sox6 (59% and 86%, respectively). During our experiments, both naked DNA and nucleosomes were allowed to interact with the increasing amounts of Sox. Here, the Sox amount used for the analysis of its binding to NCP was ∼3-fold higher than used in the naked DNA experiments. Then the samples were used for ^•^OH radical footprinting. As shown in Figure 3A, upon increasing the Sox amount in the reaction mixture, a very clear protection of all three Sox recognition sequences is observed on the naked DNA. Protection is also observed at Sox-binding sequences in histone-wrapped mutated 601 DNA (Figure 3A, insets). Albeit, the protection at SHL2 and SHL4 is not so well visible. Here, the protected sites coincide with the maximal cleavage of the DNA in the nucleosome. Of note, a very specific footprinting of Sox is detected at the nucleosome dyad, where the four middle DNA bands exhibit a strong decrease in the intensity compared to the flanking bands (see the magnified recognition sequence footprint as well as their quantification at the right inset of Figure 3A). This Sox-protected ^•^OH cleavage pattern is very similar to the one of globular linker histone H1 domain in the H1-bound nucleosome^3,5,60^ (also see Figure S6), which implies that the global 3D organization of the Sox–dyad complex is analogous to the complex formed between the globular domain of H1 and the nucleosome dyad.^3,5,60^

### UV Laser Footptinting Validates That DNA Shape Reading Is Realized at SHL2 and SHL4 but Not at the Dyad

To analyze the local DNA changes occurring at the minor groove of nucleosomal DNA, we performed UV laser footptinting of Sox-bound mutated 601 DNA in free and histone-wrapped states. UV laser footprinting measures the UV laser-induced alterations in the nucleotide photoreactivity,^61^ which could affect the spectrum and the amount of DNA lesions. Since binding of Sox to DNA alters the local structure of Sox recognition sequence, it should lead to changes in the spectrum of lesions. Such lesions are extremely sensitive to the local DNA structure and can easily be mapped by alkali or enzymatic DNA strand cleavage, followed by electrophoresis under denaturing conditions at the single nucleotide resolution.^62^ Since a single nano- or picosecond laser pulse is used for irradiation, the generation of the lesions is achieved in an interval of time, which is shorter than the conformational transitions of the protein:DNA complex.^63^ So, the laser footprinting is taking a snapshot of the complex structure while recording the Sox-induced structural signature.^63^ We used this method successfully in the past for mapping productive protein:DNA interactions.^14,64^ Followingly, we mapped UV laser-specific biphotonic 8-OxoG lesions by using Fpg glycosylase (formamidopyrimidine [fapy]-DNA glycosylase). These lesions are observed on a GG sequence, located right after the 5′-ACAA-3′, complementary to Sox cognate 5′-TTGT-3′ (Figure S7A,B and the Methods section). As expected, with the increase in Sox concentration, the disappearance of 8-oxoG bands is traced in the mutated 601 (601-SHL024) DNA constructs, indicating a deformation at the Sox cognate sequence (Figures 3B and S7C). In the case of the 601-SHL024 nucleosome, the same behavior was observed at SHL2 and SHL4, while at the dyad, no footprint was spotted (Figure 3B). Also, apparent dissociation constant (*K*_d_^app^) values of specific Sox binding to its target sequence (Table 2) were evaluated by Sox titration footprinting and gel quantification (Figures 3B (right) and S8). Interestingly, the measured apparent binding affinities for SHL2 and SHL4 nucleosomes were only 3–4 times higher than in naked DNA. The higher binding affinity of SHL2 compared to SHL4 in nucleosomes, together with the absence of specific binding at the dyad, is fully consistent with the data obtained by our computational approach. Important to note that this apparent (measured) dissociation constant (*K*_d_^app^) must not be associated with the true dissociation *K*_d_. Indeed, the two orders of magnitude higher concentrations (*R* = 50 nM) of the constant component (DNA or nucleosomes) with respect to the true *K*_d_ < 1 nM,^17^ as well as the unavoidable presence of several lower-affinity binding sites per ∼200 bp DNA fragment (due binding motif degeneracy) precluded the true *K*_d_ determination under our experimental conditions (see ref (65)). Strikingly, identical footprinting profiles were observed when the Sox cognate sequence was incorporated at the dyad, SHL2, and SHL4 separately (Figures S5, S7, and S8). We should also note that our computational results for Sox11 were reproduced when we performed our DIM protocol with a structural model of Sox6, which endorses the general applicability of our findings (Methods section; Figure S9).

**Table 2:** Apparent Dissociation Constants (*K*_d_^app^) of Sox6 Binding to Naked and Nucleosomal DNA, Representing the Concentrations of Sox6 at the Half Maximum Signal Intensity Change, Extrapolated by the Least-Squares Fitting Procedure of Data in Figures 3B (Right) and S8

SHL region	DNA *K*_d_^app^ (nM)	Nuc *K*_d_^app^ (nM)
SHL0	100	a
SHL2	136	374
SHL4	180	490
3x SHL0	198	a
3x SHL2	275	550
3x SHL4	245	680

a Denotes the absence of binding to the target sequence.

## Discussion

Our work is based on the hypothesis that PTFs follow a universal binding mechanism independent of the strand-positioning of their cognate sequences. So far, several studies presented different SHL bindings of Sox when its cognate motif faces intergyre. To complement this view, we focused on the solvent-facing binding mode of Sox. Accordingly, we found that, in this mode, Sox can comfortably fit in the DNA gyre and thus will not induce major conformational changes. We also showed that among the probed SHL0/2/4 sites, Sox base and shape reading mechanisms can be realized efficiently only at SHL2. This is in line with what was observed by Zhu et al., where, among many SHLs, Sox always selected SHL2.^20^

**Figure 4:**
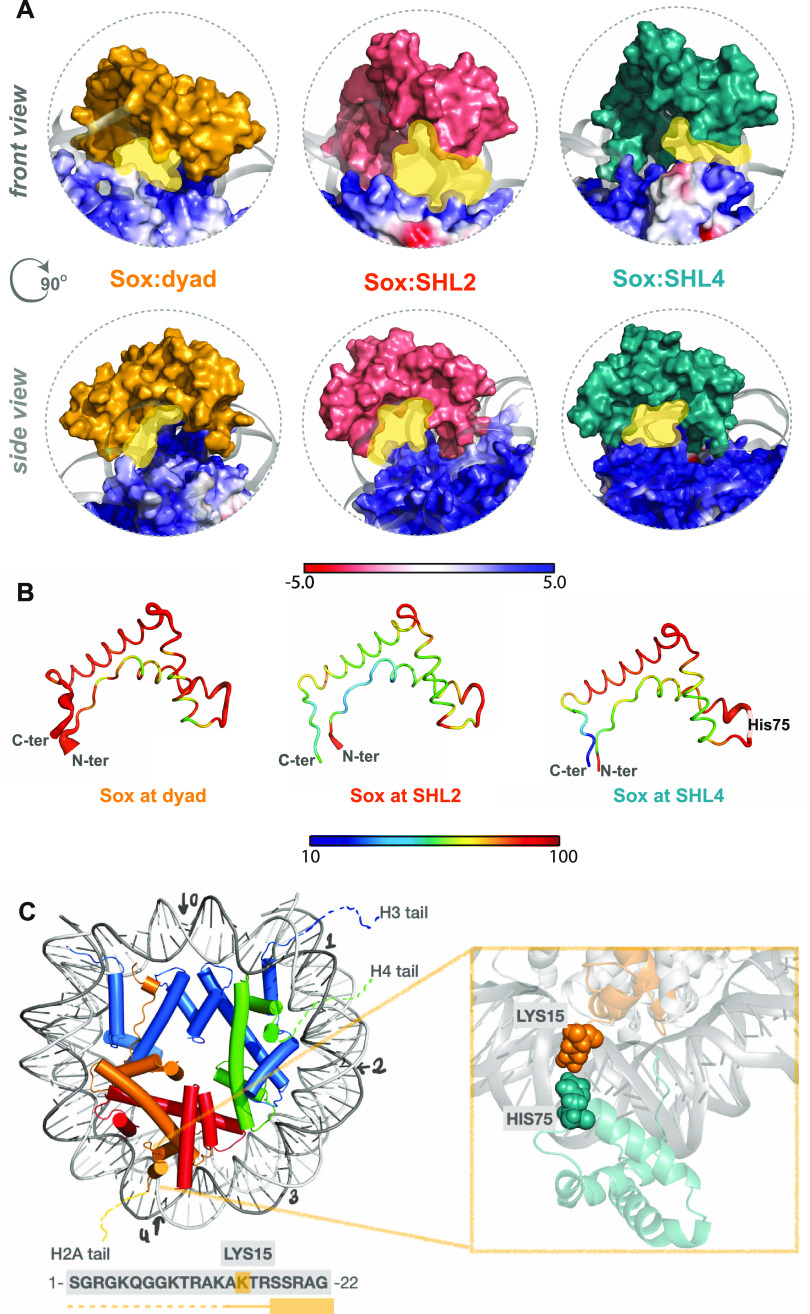
(A) SHL2 is transparent to Sox binding. Sox is far enough from the basic histones for not to feel them only at the SHL2 binding site. The nucleosomal DNAs are demonstrated as a cartoon and colored in dark gray. Histones are shown as surface and colored according to their electrostatic potentials. Here, red represents negatively charged (−5) regions and blue represents positively charged (+5) regions. (B) Sox binding is the most stable at SHL2. Sox thermal fluctuations deduced from the MD simulations of Sox11:dyad (left), Sox11:SHL2 (middle), and Sox11:SHL4 (right) are colored according to their temperature factors. Blue and red colors depict the most rigid and the most flexible regions, respectively. The cartoon thickness linearly scales with the degree of thermal fluctuations (averaged over *N* = 600 conformations). (C) Among the probed SHLs (marked with arrows), at SHL4 the N-terminal tail of H2A forms stable interactions with the nucleosomal DNA. As a result, Lys15 of H2A and His75 of Sox face each other, even in the most Sox-binding-complementary binding mode (with P-RMSD = 1.34 Å, minor groove width = 18.5 Å, where Sox-DNA fingerprint interactions were observed for critical Sox amino acids, except for Asn54).

As we move away from the dyad toward the entry/exit sites of the nucleosome, histone–DNA interaction strength gradually decreases. This mechanistic property lowers the energy barrier for histone–DNA detachment during nucleosome sliding or unwrapping.^66^ If Sox:NCP recognition would have been only a function of histone–DNA interactions, the SHL4 site should have turned out to be the most “bendable” site (as it is the closest one to the DNA entry/exit site). To understand why this is not the case, we investigated Sox:histone interactions. As a result, we uncovered that only at SHL2, the core domain of Sox does not establish any contact with the surrounding histones (Table S2). Strikingly, at SHL4, the same Sox domain forms the most histone contacts. This observation is directly reflected in the electrostatic properties of each binding site: at SHL2, Sox’s globular HMG domain is far enough not to directly feel the basic nature of histones (Figure 4A). This leads to a stable Sox11 (with the least thermal fluctuations) at SHL2 (Figure 4B). In the case of SHL4, Lys15 from the H2A N-terminal tail interferes with the conserved His75 of Sox11, resulting in a local destabilization at the tip of the Sox (Figure 4B,C). So, at SHL4, Sox binding could not induce enough perturbation to lower the energy barrier for detaching H2A’s structured N-tail from nucleosomal DNA. This is in line with a recent computational study utilizing discrete stochastic simulations, where a stable binding of PTF to the nucleosomal DNA was found to be compensated by the weakening in histone–DNA interactions.^67,68^ The same hypothesis was also formulated by Tan and Takada.^51^

Stemming from these observations, we hypothesize that, even though it harbors tighter histone–DNA interactions, SHL2 can accommodate specific Sox binding since there would be no interference of Sox with histones and its tails (H3 and H4) on this site (Figure 4C). Though, this was not the case for the intergyre-facing binding at SHL2, where H4 N-tail displacement was observed upon Sox binding,^45^ another indication of strand-positioning dependence of Sox binding. To clarify the exact roles of histone tails in intergyre-facing Sox binding, at the atomistic scale, though, more detailed experiments should be carried out. In this work, we did not consider the computational investigation of this phenomenon due to our concerns about the accountability of available force fields to address the intricate tail–protein–DNA interaction dynamics.^52−54,69^ Further experiments would be also needed to clarify whether 1.3 times better affinity at SHL2, compared to SHL4, could correspond to the energetic contribution of base-specific reading. To explore this experimentally at the atomistic scale, NMR would be the best-suiting approach. However, for this, we would need to wait until the selective nucleotide labeling in NMR experiments is established, which could alleviate the size and geometry limitations posed by the nucleosomal DNA.^70,71^

Over the various sites where nucleosomal DNA and histones are touching, the dyad contains the strongest histone–DNA interactions.^66^ This explains the inability of Sox to bend/open its binding site at the dyad, where only nonspecific binding is observed, both by us and by other groups.^48,51^ The fact that Sox ^•^OH radical footprinting at the dyad is very similar to the one of H1 globular domain at the dyad suggests that different HMG proteins could be able to efficiently bind in vivo to the dyad without requiring the presence of recognition.^5,60^ Thus, globular H1 domain-like dimensions of Sox HMG could be sufficient for the nonspecific binding of HMG proteins to the dyad. This could explain why the nonspecific and highly abundant HMGB1/B2 chromatin proteins and the linker histone would have a shared structural role in organizing linker DNA in the nucleosome,^72^ while PTFs of different shapes, such as Oct4, were never observed as a nonspecific nucleosome binder.^51^

To explore the molecular mechanism of Sox binding, we developed a DIM approach. In this approach, we used several cycles of classical MD to explore the thermal fluctuation ranges spanned by free and Sox-bound nucleosomes. We wanted to stick to unbiased simulations here due to the similar force field concerns we had for addressing histone tail dynamics. We also made use of data-driven modeling to place Sox at its binding spot in an accurate manner. This stepwise modeling procedure, together with the exclusion of histone tails, gave us the opportunity to reach convergence in rather short time scales. To further save compute time, we incorporated all three Sox binding sequences into 601 at once. The relevance of this was underscored by the identical footprinting profiles observed when Sox cognate sequences were incorporated separately at dyad, at SHL2, and at SHL4 or at once. Also, to access a wider sampling space, we pooled our replica simulations in our analyses. The analysis tools we used were discriminative enough to explain the determinants of high-affinity Sox binding, which expands on the evaluation of base-specific hydrogen bonding, minor groove width, and P-RMSD profiles, as well as the thermal fluctuations of bound Sox. The latter two were also outlined as major determinants in high-affinity Sox binding by Malaga Gadea and Nikolova.^48^ Next to these, with our work, we also add the strand placement of Sox cognate sequence as a new parameter in explaining high-affinity binding spots for Sox. As a final note, the transferability of ours and other findings obtained on the unnatural nucleosomal DNA sequences to the natural ones is yet to be explored.

In summary, our in silico and experimental observations in combination reveal that the binding of Sox is strongly nucleosomal-context-dependent, where not only histone–DNA interactions but also Sox–histone interactions dictate the binding capacity of Sox. A similar mechanism could be valid for other PTFs, which could be researched with the DIM protocol proposed in this work.

## Methods

### Dynamic Integrative Modeling

To explore Sox:nucleosome interactions (both for Sox11 and Sox6), we performed a series of modeling and MD simulation cycles, making up our dynamic integrative modeling (DIM) approach. Our DIM protocol is composed of three steps (Figure S11): (Step 1) Inserting the Sox cognate sequence into the 601 Widom DNA sequence (PDB id: 3LZ0(47)) at different SHLs, i.e., SHL0 (dyad), SHL2, and SHL4, followed by their MD simulations (free mutated nucleosome). (Step 2) Isolating the Sox-binding-compatible nucleosome conformer of each SHL to model Sox-bound nucleosomes. (Step 3) Simulating the constructed Sox-bound nucleosome structures. We also simulated the Sox11:(free)DNA complex (PDB id: 6T78(45)) and the free NCAP-SELEX nucleosome (PDB id: 6T79(45)) to serve as a reference. The technical details of each step are provided below.

(Step 1) The MD simulations of the 601 Widom DNA sequence: We inserted a special sequence bearing the Sox consensus sequence 5′-GG*ACAA*TGGAGG-3′ at dyad, SHL2, and SHL4 by using 3DNA.^73^ The mutated sequence corresponds to (−4th)–7th, 16th–27th, and 37th–48th nucleotides in the forward DNA chain, leading to: 5′-ATCAGAATCCCGGTGCCGAGGCCGCTCAATTGGTCGTAGACAGCTCTAGCACCGCTTAAACGCACGTA*GGACAATGGAGG*CGCGTTTT*GGACAATGGAGG*CATTACTCC*GGACAATGGAGG*CACGTGTCAGATATATACATCGA-3′

(Steps 2 and 3) The modeling and simulation of the Sox:NCP complexes: The free nucleosome conformers, reflecting the lowest P-RMSD to Sox-bound-DNA conformation for each binding site, were. Sox11 isolated from Sox11:DNA complex (PDB id: 6T78(45)) was located at three binding sites by template-based modeling. The initial crude Sox:nucleosome complexes were refined within HADDOCK 2.2,^55,56^ while imposing the critical Sox:DNA interactions as restraints.^74^ Here, we imposed the crystal Sox11:DNA distances, measured between 56th, 57th (FM) residues of Sox11 and 0th, 20th, and 41st nucleotides (forward strand), respectively. The best HADDOCK models were then subjected to two parallel MD simulations. In the end, the conformations with the lowest P-RMSDs were isolated and kept as final Sox11-bound nucleosome structures (for Sox:dyad, Sox:SHL2, and Sox:SHL4). The same procedure was carried out with the homology model of Sox6.

### Molecular Dynamics Simulations and Analysis Protocols

All of the MD simulations were performed with the GROMACS simulation packages (Gromacs 5.1.4, Gromacs 2019, and Gromacs 2020.4)^75^ under the effect of the Amber ff14SB (protein) and Parmbsc1 (DNA) force field.^76^ We used TIP3P as a water model. The NaCl concentration was kept at 0.15 M. A dodecahedron simulation box was used while having a minimum distance of 12 Å between the biological molecule and the edges of the simulation box. We used the PME method to address electrostatic interactions (with 1.2 nm nonbonded cutoff) and Lennard Jones potential to account for the van der Waals interactions (with 1.2 nm nonbonded cutoff). The temperature was kept at 310 K throughout the simulation. We carried out two replica simulations of each system, specifically of free 601 nucleosome (PDB id: 3LZ0) (2 × 1 μs), free NCAP-SELEX nucleosome (PDB id: 6T79) (2 × 0.5 μs), Sox11:DNA complex (PDB id: 6T78) (2 × 0.5 μs), and Sox11-bound nucleosome models, i.e., Sox11:dyad (2 × 0.5 μs), Sox11:SHL2 (2 × 0.5 μs), and Sox11:SHL4 (2 × 0.5 μs). We also carried out one simulation for each Sox6-bound nucleosome model, i.e., Sox6:dyad (1 × 0.5 μs), Sox6:SHL2 (1 × 0.5 μs), and Sox6:SHL4 (1 × 0.5 μs). The homology model of Sox6 was generated with Modeller.^77^ The complete list of our simulations is provided in Table S3.

Before running the simulations, complexes were minimized by using the steepest descent algorithm in the vacuum. Then, they were solvated in TIP3P water, and the concentration was kept at 0.15 M by adding NaCl to the system (460 Na^+^, 240 Cl^–^ for the free 601 nucleosome; 421 Na^+^, 201 Cl^–^ for the free NCAP-SELEX nucleosome, 49 Na^+^, 38 Cl^–^ for Sox11:DNA; 427 Na^+^, 218 Cl^–^ for Sox11:dyad; 438 Na^+^ and 229 Cl^–^ for Sox11:SHL2; 477 Na^+^ and 268 Cl^–^ for Sox11:SHL4; 396 Na^+^ and 184 Cl^–^ for Sox6:dyad; 418 Na^+^ and 206 Cl^–^ for Sox6:SHL2; 438 Na^+^ and 226 Cl^–^ for Sox6:SHL4). The number of ions was added to the topology files accordingly, and then the solvated systems were minimized. The systems were relaxed for 20 ps at 310 K under the constant volume. To generate replicas, random seeds were changed. Then, another 20 ps MD simulations were performed under constant pressure at 1 bar. Finally, position restraints were released gradually from 1000 to 100, 100 to 10, and 10 to 0. The integration time step was set to 2 fs. For the analysis, coordinate files were recorded every 0.5 ns. The initial 200 ns of all simulations (except for the free 601 nucleosome) were set as the equilibration time and discarded before the analysis stage. The equilibration time for 601 free nucleosome was set as 350 ns.

At the end of MD simulations, minor groove widening and P-RMSD (root-mean-square deviations of the DNA phosphorus atoms) metrics were calculated over all of the conformers. Minor groove widths were measured with 3DNA.^73^ P-RMSD values were computed over the phosphorus atoms of seven nucleotides involving the Sox recognition sequence (5′-GACAATG-3′). The reference seven nucleotides correspond to (−3rd)–3rd, 17th–23rd, and 38th–44th nucleotide positions at dyad, SHL2, and SHL4, respectively. During all of these measurements, the DNA of Sox11:DNA complex (PDB id: 6T78(45)) was taken as a reference. The fitting and P-RMSD computations were performed with Profit (Martin, A.C.R., http://www.bioinf.org.uk/software/profit/). The individual fitting profiles of each simulation are provided in Figure S10.

Molecular interaction profiles of complexes were calculated in each replica simulation by using the Interfacea Python library (https://github.com/JoaoRodrigues/interfacea).^78^ This library was used to provide the non-covalent hydrophobic, ionic interactions, and h-bonds for every coordinate file. From these output files, we extracted the interactions between essential Sox amino acids (Arg51, Asn54, Phe56, Met57, Tyr118) and DNA. We isolated the base-specific interactions among all interactions, which are the interactions between the Sox protein and the respective DNA bases. In the barcode plots, each vertical line indicated the presence of a given interaction for a given time. These graphs were plotted in MATLAB R2022B.^79^

### Sox6 HMG Domain Cloning and Purification

The HMG domain of the human Sox6 (618–697 amino acids) gene was cloned in the pET28b vector in between *Nde*I and *Xho*I restriction sites. The N terminal His-tagged Sox6 HMG domain was produced in *Escherichia coli* BL21(DE3) pLYsS cells. Briefly, 200 ng of the plasmid was used to transform it into *E. coli* cells; after transformation, bacteria were platted on Luria–Bertani (LB) agar plates supplemented with kanamycin and chloramphenicol and incubated overnight at 37 °C. Single colonies were added to 3 mL of an LB medium (kanamycin + chloramphenicol) for 12–16 h at 37 °C under shaking at 200 rpm speed. Overall, 1 mL of amplified bacteria was added to 300 mL of LB (kanamycin + chloramphenicol) and left overnight at 37 °C and 200 rpm. For each liter of LB (kanamycin + chloramphenicol) needed, 10 mL of transformed bacteria were added. After 3 h of incubation, OD at 600 nm was measured. If the OD600 was comprised between 0. 5 and 0.6, bacteria were induced with 0.2 mM isopropyl-β-d-thiogalactopyranoside (IPTG) at 37 °C for 3–4 h at 200 rpm. After induction, bacteria were pelleted at 5000*g* for 20 min at 4 °C. The recombinant human Sox6 HMG domain was purified from the supernatant of the bacterial lysate by using NiNTA resin (Complete His Tag purification Resin, Roche), followed by SP sepharose column chromatography (GE Healthcare). The purity of the purified HMG domain of Sox6 protein was analyzed by using 18% sodium dodecyl sulfate-polyacrylamide gel electrophoresis (SDS-PAGE) and stained with coomassie blue.

### Core Histone Purification

Human histones H2A, H2B, and H3 were subcloned in a pHCE vector system, and human histone H4 was subcloned in a pET15b vector system. Histones H2A, H2B, and H3 were produced in *E. coli* BL21(DE3) cells, and human H4 was produced in *E. coli* JM109(DE3) cells. Core histones were produced as N-terminal His-tagged proteins in *E. coli* cells in the absence of T7 RNA polymerase by omitting the addition of isopropyl-β-d-thiogalactopyranoside, which induces the T7 RNA polymerase production in BL21(DE3) and JM109(DE3) cells. Briefly, 200 ng of the plasmid (for each histone) was used to transform into respective *E. coli* strains. Overall, 10 colonies were inoculated into 2 L of LB broth (ampicillin final concentration 50 μg/mL) in a 5 L flask and left overnight at 37 °C and 200 rpm. Each liter of bacteria was pelleted at 5000*g* for 20 min at 4 °C. The cells producing recombinant histones were collected and disrupted by sonication in 50 mL of buffer A (50 mM Tris–HCl (pH 8.0), 500 mM NaCl, 1 mM phenylmethylsulfonyl fluoride (PMSF), and 5% glycerol). After centrifugation (27,216*g*; 20 min; 4 °C), the pellet containing His-tagged histones as insoluble forms was resuspended in 50 mL of buffer A containing 7 M guanidine hydrochloride. After centrifugation (27,216*g*; 20 min; 4 °C), the supernatants containing the His-tagged histones were combined with NiNTA resin (complete His Tag purification Resin, Roche) (1 mL of NiNTA per 1 L of bacteria) and were mixed by rotation for 1 h at 4 °C. The agarose beads were packed into an Econo-column (Bio-Rad) and were then washed with 100 mL of buffer B (50 mM Tris–HCl (pH 8.0), 500 mM NaCl, 6 M urea, 5 mM imidazole, and 5% glycerol). The His-tagged histones were eluted by a 100 mL linear gradient of imidazole from 5 to 500 mM in buffer B, and the samples were dialyzed against buffer C (5 mM Tris–HCl (pH 7.5) and 2 mM 2-mercaptoethanol).

The N-terminal 6x His tags were removed from the histones by thrombin protease (GE Healthcare) treatments using 1 unit per 1 mg of protein for 3–5 h at 4 °C. The removal of the His tags was confirmed by SDS-16% polyacrylamide gel electrophoresis (PAGE); the recombinant histones without the His tag migrated faster than the His-tagged histones. After uncoupling of the His tag, each histone was subjected to Resource S column chromatography (GE Healthcare). The column was washed with buffer D (20 mM sodium acetate (pH 5.2), 200 mM NaCl, 5 mM 2-mercaptoethanol, 1 mM ethylenediaminetetraacetic acid (EDTA), and 6 M urea), and each histone was eluted by a linear gradient of NaCl from 200 to 900 mM in buffer D. The fractions containing the pure histone were mixed and stored at −80 °C.

### Preparation of Histone Tetramers and Dimers

To prepare tetramers and dimers, human H3 and H4 and human H2A and H2B were mixed in an equimolar ratio and dialyzed overnight in HFB buffer (2 M NaCl, 10 mM Tris pH 7.4, 1 mM EDTA pH 8 and 10 mM β-mercaptoethanol). After dialysis, the supernatant containing folded tetramers and dimers were subjected to Superose 6 prep grade XK 16/70 size exclusion column (GE Healthcare) purification using HFB buffer. The major fractions containing purified tetramers and dimers were mixed. For long time storage, tetramers and dimers were mixed with NaCl-saturated glycerol to achieve the final glycerol concentration of around 15–20% and stored at −20 °C.

### Preparation of DNA Fragments

The 255 bp of 601 DNA constructs containing Sox6 consensus motif 5′-GGACAATGGAGG-3′ positioned at different places were produced by a chemical synthesis method and cloned into standard vector pEX-A by Eurofins Genomics, Germany. The positions of Sox6-binding sites in the 601 constructs are mentioned below:

Sox-SHL0 (dyad): (Sox6-binding motif located at 66 bp away from the end of nucleosomal DNA)

GCATGATTCTTAAGACCGAGTTCATCCCTTATGTGATGGACCCTATACGCGGCCGCCATCAGAATCCCGGTGCCGAGGCCGCTCAATTGGTCGTAGACAGCTCTAGCACCGCTTAAACGCACGTAGGACAATGGAGGCGCGTTTTAACCGCCAAGGGGATTACTCCCTAGTCTCCAGGCACGTGTCAGATATATACATCGATGTGCATGTATTGAACAGCGACCTTGCCGGTGCCAGTCGGATAGAATTCCGGAC

Sox6-SHL2: (Sox6-binding motif located at 46 bp away from the end of nucleosomal DNA)

GCATGATTCTTAAGACCGAGTTCATCCCTTATGTGATGGACCCTATACGCGGCCGCCATCAGAATCCCGGTGCCGAGGCCGCTCAATTGGTCGTAGACAGCTCTAGCACCGCTTAAACGCACGTACGCGCTGTCCCCCGCGTTTTGGACAATGGAGGCATTACTCCCTAGTCTCCAGGCACGTGTCAGATATATACATCGATGTGCATGTATTGAACAGCGACCTTGCCGGTGCCAGTCGGATAGAATTCCGGAC

Sox6-SHL4: (Sox6-binding motif located at 25 bp away from the end of nucleosomal DNA)

GCATGATTCTTAAGACCGAGTTCATCCCTTATGTGATGGACCCTATACGCGGCCGCCATCAGAATCCCGGTGCCGAGGCCGCTCAATTGGTCGTAGACAGCTCTAGCACCGCTTAAACGCACGTACGCGCTGTCCCCCGCGTTTTAACCGCCAAGGGGATTACTCCGGACAATGGAGGCACGTGTCAGATATATACATCGATGTGCATGTATTGAACAGCGACCTTGCCGGTGCCAGTCGGATAGAATTCCGGAC

Sox6-SHL024: (Sox6-binding motifs located at 66, 46, and 25 bp away from the end of nucleosomal DNA)

GCATGATTCTTAAGACCGAGTTCATCCCTTATGTGATGGACCCTATACGCGGCCGCCATCAGAATCCCGGTGCCGAGGCCGCTCAATTGGTCGTAGACAGCTCTAGCACCGCTTAAACGCACGTAGGACAATGGAGGCGCGTTTTGGACAATGGAGGCATTACTCCGGACAATGGAGGCACGTGTCAGATATATACATCGATGTGCATGTATTGAACAGCGACCTTGCCGGTGCCAGTCGGATAGAATTCCGGAC

All Sox6-binding motifs harboring 601 constructs were amplified using ^32^P end-labeled primers. The labeled DNA substrates were purified on 5% native acryl amide gel prior to use for nucleosome reconstitutions.

### Nucleosome Reconstitution

Nucleosome reconstitution was performed by the salt dialysis procedure. Approximately, 250 ng of a 32P-labeled DNA probe containing the Sox6-binding site and 4.5 μg of chicken erythrocyte DNA (150–200 bp) as a carrier were mixed with human histones–tetramers and dimers approximately in a 1:0.5:0.5 ratio in HFB buffer (2 M NaCl, 10 mM Tris pH 7.4, 1 mM EDTA pH 8 and 10 mM β-mercaptoethanol), respectively. The mixtures were transferred into dialysis tubing, and the reconstitution was done by dialysis against a slowly decreasing salt buffer. The NaCl concentration starts at 2 M and decreases slowly up to 500 mM NaCl. Indeed, with the help of a peristaltic pump, low salt buffer is added to the high salt buffer beaker at the rate of 1.5 mL/min for 18 h. Once finished, the dialysis bags were transferred to a 300 mM NaCl buffer and left for buffer exchange for 2 h, which was followed by final dialysis in 10 mM NaCl buffer overnight. All NaCl buffers for reconstitution include 10 mM Tris pH 7.4, 0.25 mM EDTA, 10 mM β-mercaptoethanol, and the desired amounts of NaCl.

### Sox6 HMG Domain Binding Reaction

The binding reaction of the Sox6 HMG domain on DNA or nucleosomes was carried out at 37 °C. Typically, the Sox6 HMG domain was mixed with DNA or a nucleosome (50 nM) in a 20 μL reaction containing 1× binding buffer (10 mM Tris, pH 7.4, 75 mM NaCl, 1 mM EDTA, 1 mM dithiothreitol (DTT), 100 mg/mL bovine serum albumin (BSA), 0.01% NP40 and 5% glycerol). The naked DNA was supplemented with carrier nucleosomes to a final concentration equal to those of labeled nucleosomes. The maximal Sox6 concentration was 1000 and 3000 nM in naked DNA and nucleosome samples, respectively, and the dilution step was 1.5. An aliquot of this reaction mix was used to check the formation of the Sox6 HMG domain:DNA or Sox6 HMG domain:nucleosome complex by 5% native PAGE at room temperature in 0.3× Tris–borate–EDTA (TBE) buffer. The remaining aliquots were probed by UV laser footprinting.

### Hydroxyl Radical Footprinting

Hydroxyl radical footprinting was carried out to check the strong nucleosome positioning ability of the Sox6-binding site-incorporated 255 bp of 601 constructs. The reaction was carried out in a 15 μL final reaction mixture in quencher-free buffer placed at the bottom of an Eppendorf tube. The hydroxyl radicals were generated by mixing 2.5 μL each of 2 mM FeAmSO_4_/4 mM EDTA, 0.1 M ascorbate, and 0.12% H_2_O_2_ together in a drop on the side of the reaction tube before mixing rapidly with the reaction solution. The reaction was terminated after 2 min by addition of 100 μL of a stop solution (0.1% SDS, 25 mM EDTA, 1% glycerol, and 100 mM Tris, pH 7.4), and the DNA was purified by phenol/chloroform extraction and ethanol/glycogen precipitation. The DNA was resuspended in formamide loading buffer, heated for 3 min at 80 °C, and ran along with UV laser samples on 8% denaturing gel in 1× TBE buffer. The gels were dried and exposed overnight on a phosphor imager screen. The gels were scanned on a phosphor imager and analyzed by Multi-Gauge (Fuji) software.

### UV Laser Footprinting

The UV laser-specific biphotonic lesions 8-oxoG were mapped by Fpg glycosylase, which is generated in the Sox6 cognate binding sequence upon UV laser irradiation. The samples were exposed to a single high-intensity UV laser pulse (Epulse ∼0.1 J/cm^2^), as described in previous studies.^14,80^ The DNA was then purified by phenol–chloroform and ethanol/glycogen precipitated. The purified DNA was resuspended in resuspension buffer (10 mM Tris, pH 7.4, 30 mM NaCl, 1 mM EDTA, 1 mM DTT, 100 μg/mL BSA, 0.01% NP40) and cleaved with 0.1 units of Fpg glycosylase. The DNA was lyophilized and resuspended in formamide loading buffer, heated for 3 min at 80 °C, and loaded on 8% sequencing gel in 1× TBE buffer. The gels were dried and exposed overnight on a phosphor imager screen. The screens were scanned on a phosphor imager and analyzed by Multi-Gauge (Fuji) software.

### Gel Quantification and Apparent Dissociation Constant (*K*_d_^app^) Evaluation

Gel quantifications were performed by integration of rectangles encompassing the cleavage bands of interest. Signal intensities were determined as the relative averaged intensities of the footprinted GG cleavage bands. For a given binding site, these bands were normalized to the ″internal standard″ bands, belonging to four to five other guanines within the respective DNA ladder in the absence of Sox6. The averaged and normalized relative intensities were plotted as a function of the Sox6 concentration together with the mean deviations. To evaluate the apparent dissociation constant (*K*_d_^app^), the experimental data were fitted mathematically to smoothly decaying curves by least-squares deviation procedure using MATLAB R2022B software with a biexponential function (fitexp2) providing *R*^2^ > 0.97 and RMSE < 0.05^79^ (Table 3). The apparent dissociation constant, by analogy with the true *K*_d_, was determined as the Sox6 concentration corresponding to the 1/2 level of the signal intensity change.

**Table 3:** Biexponential Function [*f*1(*x*) = *a* exp(*b* × *x*) + *c* exp(*d* × *x*)] Parameters Used in Curve Fitting

parameter	DNA-SHL024-SHL0	DNA-SHL024-SHL2	DNA-SHL024-SHL4	NUC-SHL024-SHL0	NUC-SHL024-SHL2	NUC-SHL024-SHL4
*a*	1.050	1.024	1.016	0.222	1.037	1.069
*b*	–0.0027	–0.0015	–0.0023	–6.4594 × 10^–4^	–8.163 × 10^–4^	–6.697 × 10^–4^
*c*	0.0018	1.94 × 10^–8^	0.0340	0.7894	0.0389	0.0031
*d*	0.0048	0.0156	0.0018	4.335 × 10^–5^	7.08 × 10^–4^	0.0014
*R*^2^	0.980	0.976	0.958	0.673	0.959	0.955
RMSE	0.051	0.045	0.067	0.027	0.0634	0.069

## Data Availability

All of the structural data and their analysis scripts are deposited at GitHub (https://github.com/CSB-KaracaLab/Sox-PTF). The raw gel images are submitted alongside this paper.

## Supporting Information Available

Additional computational and experimental data analysis, detailed flowchart of the computational approach, tables reporting on the simulated Sox:DNA interactions, and movies presenting the movements observed during our simulations (PDF)

## References

[ref1] van Holde K. E. The Proteins of Chromatin. I. Histones. Chromatin; Springer: New York, 1989; pp 69–180.

[ref2] Thoma F.; Koller T.; Klug A. Involvement of Histone H1 in the Organization of the Nucleosome and of the Salt-Dependent Superstructures of Chromatin. J. Cell Biol. 1979, 83, 403 10.1083/JCB.83.2.403. 387806 PMC2111545

[ref3] Syed S. H.; Goutte-Gattat D.; Becker N.; Meyer S.; Shukla M. S.; Hayes J. J.; Everaers R.; Angelov D.; Bednar J.; Dimitrov S. Single-Base Resolution Mapping of H1-Nucleosome Interactions and 3D Organization of the Nucleosome. Proc. Natl. Acad. Sci. U.S.A. 2010, 107, 9620–9625. 10.1073/PNAS.1000309107. 20457934 PMC2906896

[ref4] Meyer S.; Becker N. B.; Syed S. H.; Goutte-Gattat D.; Shukla M. S.; Hayes J. J.; Angelov D.; Bednar J.; Dimitrov S.; Everaers R. From Crystal and NMR Structures, Footprints and Cryo-Electron-Micrographs to Large and Soft Structures: Nanoscale Modeling of the Nucleosomal Stem. Nucleic Acids Res. 2011, 39, 9139 10.1093/NAR/GKR573. 21835779 PMC3241633

[ref5] Bednar J.; Garcia-Saez I.; Boopathi R.; Cutter A. R.; Papai G.; Reymer A.; Syed S. H.; Lone I. N.; Tonchev O.; Crucifix C.; Menoni H.; Papin C.; Skoufias D. A.; Kurumizaka H.; Lavery R.; Hamiche A.; Hayes J. J.; Schultz P.; Angelov D.; Petosa C.; Dimitrov S. Structure and Dynamics of a 197 Bp Nucleosome in Complex with Linker Histone H1. Mol. Cell 2017, 66, 384.e8–397.e8. 10.1016/j.molcel.2017.04.012. 28475873 PMC5508712

[ref6] Kobayashi W.; Kurumizaka H. Structural Transition of the Nucleosome during Chromatin Remodeling and Transcription. Curr. Opin. Struct. Biol. 2019, 59, 107–114. 10.1016/J.SBI.2019.07.011. 31473439

[ref7] Zhou C. Y.; Johnson S. L.; Gamarra N. I.; Narlikar G. J. Mechanisms of ATP-Dependent Chromatin Remodeling Motors. Annu. Rev. Biophys. 2016, 45, 153–181. 10.1146/ANNUREV-BIOPHYS-051013-022819. 27391925 PMC9157391

[ref8] Narlikar G. J.; Sundaramoorthy R.; Owen-Hughes T. Mechanisms and Functions of ATP-Dependent Chromatin-Remodeling Enzymes. Cell 2013, 154, 490 10.1016/J.CELL.2013.07.011. 23911317 PMC3781322

[ref9] Clapier C. R.; Iwasa J.; Cairns B. R.; Peterson C. L. Mechanisms of Action and Regulation of ATP-Dependent Chromatin-Remodelling Complexes. Nat. Rev. Mol. Cell Biol. 2017, 18, 407 10.1038/NRM.2017.26. 28512350 PMC8127953

[ref10] Wolffe A. P.; Almouzni G.; Ura K.; Pruss D.; Hayes J. J. Transcription Factor Access to DNA in the Nucleosome. Cold Spring Harbor Symp. Quant. Biol. 1993, 58, 225–235. 10.1101/SQB.1993.058.01.027. 7956033

[ref11] Beato M.; Eisfeld K. Transcription Factor Access to Chromatin. Nucleic Acids Res. 1997, 25, 3559 10.1093/nar/25.18.3559. 9278473 PMC146956

[ref12] Eisfeld K.; Candau R.; Truss M.; Beato M. Binding of NF1 to the MMTV Promoter in Nucleosomes: Influence of Rotational Phasing, Translational Positioning and Histone H1. Nucleic Acids Res. 1997, 25, 3733 10.1093/nar/25.18.3733. 9278498 PMC146933

[ref13] Vitolo J. M.; Yang Z.; Basavappa R.; Hayes J. J. Structural Features of Transcription Factor IIIA Bound to a Nucleosome in Solution. Mol. Cell. Biol. 2004, 24, 697 10.1128/MCB.24.2.697-707.2004. 14701742 PMC343799

[ref14] Lone I. N.; Shukla M. S.; Charles Richard J. L.; Peshev Z. Y.; Dimitrov S.; Angelov D. Binding of NF-KB to Nucleosomes: Effect of Translational Positioning, Nucleosome Remodeling and Linker Histone H1. PLoS Genet. 2013, 9, e1003830 10.1371/JOURNAL.PGEN.1003830. 24086160 PMC3784511

[ref15] Le Dily F. L.; Baù D.; Pohl A.; Vicent G. P.; Serra F.; Soronellas D.; Castellano G.; Wright R. H. G.; Ballare C.; Filion G.; Marti-Renom M. A.; Beato M. Distinct Structural Transitions of Chromatin Topological Domains Correlate with Coordinated Hormone-Induced Gene Regulation. Genes Dev. 2014, 28, 2151 10.1101/GAD.241422.114. 25274727 PMC4180976

[ref16] Hsu H. T.; Chen H. M.; Yang Z.; Wang J.; Lee N. K.; Burger A.; Zaret K.; Liu T.; Levine E.; Mango S. E. Recruitment of RNA Polymerase II by the Pioneer Transcription Factor PHA-4. Science 2015, 348, 1372 10.1126/SCIENCE.AAB1223. 26089518 PMC4861314

[ref17] Soufi A.; Garcia M. F.; Jaroszewicz A.; Osman N.; Pellegrini M.; Zaret K. S. Pioneer Transcription Factors Target Partial DNA Motifs on Nucleosomes to Initiate Reprogramming. Cell 2015, 161, 555 10.1016/J.CELL.2015.03.017. 25892221 PMC4409934

[ref18] Iwafuchi-Doi M.; Donahue G.; Kakumanu A.; Watts J. A.; Mahony S.; Pugh B. F.; Lee D.; Kaestner K. H.; Zaret K. S. The Pioneer Transcription Factor FoxA Maintains an Accessible Nucleosome Configuration at Enhancers for Tissue-Specific Gene Activation. Mol. Cell 2016, 62, 79–91. 10.1016/J.MOLCEL.2016.03.001. 27058788 PMC4826471

[ref19] Zaret K. S.; Lerner J.; Iwafuchi-Doi M. Chromatin Scanning by Dynamic Binding of Pioneer Factors. Mol. Cell 2016, 62, 665–667. 10.1016/J.MOLCEL.2016.05.024. 27259199 PMC4893772

[ref20] Zhu F.; Farnung L.; Kaasinen E.; Sahu B.; Yin Y.; Wei B.; Dodonova S. O.; Nitta K. R.; Morgunova E.; Taipale M.; Cramer P.; Taipale J. The Interaction Landscape between Transcription Factors and the Nucleosome. Nature 2018, 562, 76–81. 10.1038/S41586-018-0549-5. 30250250 PMC6173309

[ref21] Grossman S. R.; Engreitz J.; Ray J. P.; Nguyen T. H.; Hacohen N.; Lander E. S. Positional Specificity of Different Transcription Factor Classes within Enhancers. Proc. Natl. Acad. Sci. U.S.A. 2018, 115, E7222–E7230. 10.1073/PNAS.1804663115. 29987030 PMC6065035

[ref22] Luzete-Monteiro E.; Zaret K. S. Structures and Consequences of Pioneer Factor Binding to Nucleosomes. Curr. Opin. Struct. Biol. 2022, 75, 102425 10.1016/J.SBI.2022.102425. 35863165 PMC9976633

[ref23] Landsman D.; Bustin M. A Signature for the HMG-1 Box DNA-Binding Proteins. BioEssays 1993, 15, 539–546. 10.1002/BIES.950150807. 8135767

[ref24] Read C. M.; Cary P. D.; Crane-robinson C.; Driscoll P. C.; Norman D. G. Solution Structure of a DNA-Binding Domain from HMG1. Nucleic Acids Res. 1993, 21, 3427 10.1093/NAR/21.15.3427. 8346022 PMC331441

[ref25] Jones D. N.; Searles M. A.; Shaw G. L.; Churchill M. E.; Ner S. S.; Keeler J.; Travers A. A.; Neuhaus D. The Solution Structure and Dynamics of the DNA-Binding Domain of HMG-D from Drosophila Melanogaster. Structure 1994, 2, 609–627. 10.1016/S0969-2126(00)00063-0. 7922039

[ref26] Baxevanis A. D.; Bryant S. H.; Landsman D. Homology Model Building of the HMG-1 Box Structural Domain. Nucleic Acids Res. 1995, 23, 1019–1029. 10.1093/NAR/23.6.1019. 7731789 PMC306800

[ref27] Sutrias-Grau M.; Bianchi M. E.; Bernués J. High Mobility Group Protein 1 Interacts Specifically with the Core Domain of Human TATA Box-Binding Protein and Interferes with Transcription Factor IIB within the Pre-Initiation Complex. J. Biol. Chem. 1999, 274, 1628–1634. 10.1074/JBC.274.3.1628. 9880542

[ref28] Degryse B.; Bonaldi T.; Scaffidi P.; Müller S.; Resnati M.; Sanvito F.; Arrigoni G.; Bianchi M. E. The High Mobility Group (HMG) Boxes of the Nuclear Protein HMG1 Induce Chemotaxis and Cytoskeleton Reorganization in Rat Smooth Muscle Cells. J. Cell Biol. 2001, 152, 1197–1206. 10.1083/JCB.152.6.1197. 11257120 PMC2199202

[ref29] Bianchi M. E.; Agresti A. HMG Proteins: Dynamic Players in Gene Regulation and Differentiation. Curr. Opin. Genet. Dev. 2005, 15, 496–506. 10.1016/J.GDE.2005.08.007. 16102963

[ref30] Štros M.; Launholt D.; Grasser K. D. The HMG-Box: A Versatile Protein Domain Occurring in a Wide Variety of DNA-Binding Proteins. Cell. Mol. Life Sci. 2007, 64, 2590–2606. 10.1007/S00018-007-7162-3. 17599239 PMC11136187

[ref31] Balliano A.; Hao F.; Njeri C.; Balakrishnan L.; Hayes J. J. HMGB1 Stimulates Activity of Polymerase β on Nucleosome Substrates. Biochemistry 2017, 56, 647–656. 10.1021/acs.biochem.6b00569. 28098985 PMC5679249

[ref32] Palozola K. C.; Lerner J.; Zaret K. S. A Changing Paradigm of Transcriptional Memory Propagation through Mitosis. Nat. Rev. Mol. Cell Biol. 2019, 20, 55–64. 10.1038/s41580-018-0077-z. 30420736 PMC6557398

[ref33] Julian L. M.; McDonald A. C.; Stanford W. L. Direct Reprogramming with SOX Factors: Masters of Cell Fate. Curr. Opin. Genet. Dev. 2017, 46, 24–36. 10.1016/J.GDE.2017.06.005. 28662445

[ref34] Frum T.; Murphy T. M.; Ralston A. HIPPO Signaling Resolves Embryonic Cell Fate Conflicts during Establishment of Pluripotency in Vivo. eLife 2018, 7, e42298 10.7554/ELIFE.42298. 30526858 PMC6289571

[ref35] Tiwari N.; Tiwari V. K.; Waldmeier L.; Balwierz P. J.; Arnold P.; Pachkov M.; Meyer-Schaller N.; Schübeler D.; van Nimwegen E.; Christofori G. Sox4 Is a Master Regulator of Epithelial-Mesenchymal Transition by Controlling Ezh2 Expression and Epigenetic Reprogramming. Cancer Cell 2013, 23, 768–783. 10.1016/J.CCR.2013.04.020. 23764001

[ref36] Lourenço A. R.; Coffer P. J. SOX4: Joining the Master Regulators of Epithelial-to-Mesenchymal Transition?. Trends Cancer 2017, 3, 571–582. 10.1016/J.TRECAN.2017.06.002. 28780934

[ref37] Malarkey C. S.; Churchill M. E. A. The High Mobility Group Box: The Ultimate Utility Player of a Cell. Trends Biochem. Sci. 2012, 37, 553–562. 10.1016/J.TIBS.2012.09.003. 23153957 PMC4437563

[ref38] Rohs R.; Jin X.; West S. M.; Joshi R.; Honig B.; Mann R. S. Origins of Specificity in Protein-DNA Recognition. Annu. Rev. Biochem. 2010, 79, 233–269. 10.1146/ANNUREV-BIOCHEM-060408-091030. 20334529 PMC3285485

[ref39] Klaus M.; Prokoph N.; Girbig M.; Wang X.; Huang Y. H.; Srivastava Y.; Hou L.; Narasimhan K.; Kolatkar P. R.; Francois M.; Jauch R. Structure and Decoy-Mediated Inhibition of the SOX18/Prox1-DNA Interaction. Nucleic Acids Res. 2016, 44, 3922 10.1093/NAR/GKW130. 26939885 PMC4856986

[ref40] Jauch R.; Ng C. K. L.; Narasimhan K.; Kolatkar P. R. The Crystal Structure of the Sox4 HMG Domain–DNA Complex Suggests a Mechanism for Positional Interdependence in DNA Recognition. Biochem. J. 2012, 443, 39–47. 10.1042/BJ20111768. 22181698

[ref41] Palasingam P.; Jauch R.; Ng C. K. L.; Kolatkar P. R. The Structure of Sox17 Bound to DNA Reveals a Conserved Bending Topology but Selective Protein Interaction Platforms. J. Mol. Biol. 2009, 388, 619–630. 10.1016/J.JMB.2009.03.055. 19328208

[ref42] Murphy E. C.; Zhurkin V. B.; Louis J. M.; Cornilescu G.; Clore G. M. Structural Basis for SRY-Dependent 46-X,Y Sex Reversal: Modulation of DNA Bending by a Naturally Occurring Point Mutation. J. Mol. Biol. 2001, 312, 481–499. 10.1006/JMBI.2001.4977. 11563911

[ref43] Werner M. H.; Huth J. R.; Gronenborn A. M.; Clore G. M. Molecular Basis of Human 46X,Y Sex Reversal Revealed from the Three-Dimensional Solution Structure of the Human SRY-DNA Complex. Cell 1995, 81, 705–714. 10.1016/0092-8674(95)90532-4. 7774012

[ref44] Hou L.; Srivastava Y.; Jauch R. Molecular Basis for the Genome Engagement by Sox Proteins. Semin. Cell Dev. Biol. 2017, 63, 2–12. 10.1016/J.SEMCDB.2016.08.005. 27521520

[ref45] Dodonova S. O.; Zhu F.; Dienemann C.; Taipale J.; Cramer P. Nucleosome-Bound SOX2 and SOX11 Structures Elucidate Pioneer Factor Function. Nature 2020, 580, 669–672. 10.1038/s41586-020-2195-y. 32350470

[ref46] Michael A. K.; Grand R. S.; Isbel L.; Cavadini S.; Kozicka Z.; Kempf G.; Bunker R. D.; Schenk A. D.; Graff-Meyer A.; Pathare G. R.; Weiss J.; Matsumoto S.; Burger L.; Schübeler D.; Thomä N. H. Mechanisms of OCT4-SOX2 Motif Readout on Nucleosomes. Science 2020, 368, 1460–1465. 10.1126/SCIENCE.ABB0074. 32327602

[ref47] Vasudevan D.; Chua E. Y. D.; Davey C. A. Crystal Structures of Nucleosome Core Particles Containing the ‘601’ Strong Positioning Sequence. J. Mol. Biol. 2010, 403, 1–10. 10.1016/J.JMB.2010.08.039. 20800598

[ref48] Malaga Gadea F. C.; Nikolova E. N. Structural Plasticity of Pioneer Factor Sox2 and DNA Bendability Modulate Nucleosome Engagement and Sox2-Oct4 Synergism. J. Mol. Biol. 2023, 435, 167916 10.1016/J.JMB.2022.167916. 36495920 PMC10184184

[ref49] Huertas J.; MacCarthy C. M.; Schöler H. R.; Cojocaru V. Nucleosomal DNA Dynamics Mediate Oct4 Pioneer Factor Binding. Biophys. J. 2020, 118, 2280–2296. 10.1016/J.BPJ.2019.12.038. 32027821 PMC7202942

[ref50] Huertas J.; Schöler H. R.; Cojocaru V. Histone Tails Cooperate to Control the Breathing of Genomic Nucleosomes. PLoS Comput. Biol. 2021, 17, e1009013 10.1371/JOURNAL.PCBI.1009013. 34081696 PMC8174689

[ref51] Tan C.; Takada S. Nucleosome Allostery in Pioneer Transcription Factor Binding. Proc. Natl. Acad. Sci. U.S.A. 2020, 117, 20586–20596. 10.1073/PNAS.2005500117. 32778600 PMC7456188

[ref52] Duong V. T.; Chen Z.; Thapa M. T.; Luo R. Computational Studies of Intrinsically Disordered Proteins. J. Phys. Chem. B 2018, 122, 10455–10469. 10.1021/ACS.JPCB.8B09029. 30372613 PMC6249069

[ref53] Rabdano S. O.; Shannon M. D.; Izmailov S. A.; Gonzalez Salguero N.; Zandian M.; Purusottam R. N.; Poirier M. G.; Skrynnikov N. R.; Jaroniec C. P. Histone H4 Tails in Nucleosomes: A Fuzzy Interaction with DNA. Angew. Chem., Int. Ed. 2021, 60, 6480–6487. 10.1002/ANIE.202012046. PMC7994933 33522067

[ref54] Musselman C. A.; Kutateladze T. G. Visualizing Conformational Ensembles of the Nucleosome by NMR. ACS Chem. Biol. 2022, 17, 495–502. 10.1021/acschembio.1c00954. 35196453 PMC9089449

[ref55] Dominguez C.; Boelens R.; Bonvin A. M. J. J. HADDOCK: A Protein-Protein Docking Approach Based on Biochemical or Biophysical Information. J. Am. Chem. Soc. 2003, 125, 1731–1737. 10.1021/JA026939X. 12580598

[ref56] Van Zundert G. C. P.; Rodrigues J. P. G. L. M.; Trellet M.; Schmitz C.; Kastritis P. L.; Karaca E.; Melquiond A. S. J.; Van Dijk M.; De Vries S. J.; Bonvin A. M. J. J. The HADDOCK2.2 Web Server: User-Friendly Integrative Modeling of Biomolecular Complexes. J. Mol. Biol. 2016, 428, 720–725. 10.1016/J.JMB.2015.09.014. 26410586

[ref57] Churchill M. E. A.; Hayes J. J.; Tullius T. D. Detection of Drug Binding to DNA by Hydroxyl Radical Footprinting. Relationship of Distamycin Binding Sites to DNA Structure and Positioned Nucleosomes on 5S RNA Genes of Xenopus. Biochemistry 1990, 29, 6043–6050. 10.1021/BI00477A023. 1696501

[ref58] Dixon W. J.; Hayes J. J.; Levin J. R.; Weidner M. F.; Dombroski B. A.; Tullius T. D. Hydroxyl Radical Footprinting. Methods in Enzymology; Elsevier B.V., 1991; Vol. 208, pp 380–413. 1664026 10.1016/0076-6879(91)08021-9

[ref59] Jagannathan I.; Hayes J. J. Hydroxyl Radical Footprinting of Protein–DNA Complexes. DNA–Protein Interactions, Methods in Molecular Biology; Humana Press, 2009; Vol. 543, pp 57–71. 10.1007/978-1-60327-015-1_5 19378159

[ref60] Garcia-Saez I.; Menoni H.; Boopathi R.; Shukla M. S.; Soueidan L.; Noirclerc-Savoye M.; Le Roy A.; Skoufias D. A.; Bednar J.; Hamiche A.; Angelov D.; Petosa C.; Dimitrov S. Structure of an H1-Bound 6-Nucleosome Array Reveals an Untwisted Two-Start Chromatin Fiber Conformation. Mol. Cell 2018, 72, 902.e7–915.e7. 10.1016/J.MOLCEL.2018.09.027. 30392928

[ref61] Douki T.; Angelov D.; Cadet J. UV Laser Photolysis of DNA: Effect of Duplex Stability on Charge-Transfer Efficiency. J. Am. Chem. Soc. 2001, 123, 11360–11366. 10.1021/JA016426A. 11707110

[ref62] Angelov D.; Khochbin S.; Dimitrov S. UV Laser Footprinting and Protein-DNA Crosslinking. Methods Mol. Biol. 1999, 119, 481–495. 10.1385/1-59259-681-9:481. 10804534

[ref63] Pashev I. G.; Dimitrov S. I.; Angelov D. Crosslinking Proteins to Nucleic Acids by Ultraviolet Laser Irradiation. Trends Biochem. Sci. 1991, 16, 323–326. 10.1016/0968-0004(91)90133-G. 1835191

[ref64] Angelov D.; Novakov E.; Khochbin S.; Dimitrov S. Ultraviolet Laser Footprinting of Histone H1°–Four-Way Junction DNA Complexes. Biochemistry 1999, 38, 11333–11339. 10.1021/bi9905260. 10471283

[ref65] Jarmoskaite I.; Alsadhan I.; Vaidyanathan P. P.; Herschlag D. How to Measure and Evaluate Binding Affinities. eLife 2020, 9, e57264 10.7554/ELIFE.57264. 32758356 PMC7452723

[ref66] Bowman G. D.; Poirier M. G. Post-Translational Modifications of Histones That Influence Nucleosome Dynamics. Chem. Rev. 2015, 115, 2274–2295. 10.1021/cr500350x. 25424540 PMC4375056

[ref67] Mondal A.; Mishra S. K.; Bhattacherjee A. Kinetic Origin of Nucleosome Invasion by Pioneer Transcription Factors. Biophys. J. 2021, 120, 5219–5230. 10.1016/J.BPJ.2021.10.039. 34757077 PMC8715162

[ref68] Felipe C.; Shin J.; Kolomeisky A. B. How Pioneer Transcription Factors Search for Target Sites on Nucleosomal DNA. J. Phys. Chem. B 2022, 126, 4061–4068. 10.1021/ACS.JPCB.2C01931. 35622093

[ref69] Ghoneim M.; Fuchs H. A.; Musselman C. A. Histone Tail Conformations: A Fuzzy Affair with DNA. Trends Biochem. Sci. 2021, 46, 564–578. 10.1016/J.TIBS.2020.12.012. 33551235 PMC8195839

[ref70] van Emmerik C. L.; van Ingen H. Unspinning Chromatin: Revealing the Dynamic Nucleosome Landscape by NMR. Prog. Nucl. Magn. Reson. Spectrosc. 2019, 110, 1–19. 10.1016/J.PNMRS.2019.01.002. 30803691

[ref71] Abramov G.; Velyvis A.; Rennella E.; Wong L. E.; Kay L. E. A Methyl-TROSY Approach for NMR Studies of High-Molecular-Weight DNA with Application to the Nucleosome Core Particle. Proc. Natl. Acad. Sci. U.S.A. 2020, 117, 12836–12846. 10.1073/PNAS.2004317117. 32457157 PMC7293644

[ref72] Ura K.; Nightingale K.; Wolffe A. P. Differential Association of HMG1 and Linker Histones B4 and H1 with Dinucleosomal DNA: Structural Transitions and Transcriptional Repression. EMBO J. 1996, 15, 4959 10.1002/j.1460-2075.1996.tb00876.x. 8890169 PMC452233

[ref73] Lu X. J.; Olson W. K. 3DNA: A Versatile, Integrated Software System for the Analysis, Rebuilding and Visualization of Three-Dimensional Nucleic-Acid Structures. Nat. Protocols 2008, 3, 1213–1227. 10.1038/nprot.2008.104. 18600227 PMC3065354

[ref74] Karaca E.; Rodrigues J. P. G. L. M.; Graziadei A.; Bonvin A. M. J. J.; Carlomagno T. M3: An Integrative Framework for Structure Determination of Molecular Machines. Nat. Methods 2017, 14, 897–902. 10.1038/NMETH.4392. 28805795

[ref75] Van Der Spoel D.; Lindahl E.; Hess B.; Groenhof G.; Mark A. E.; Berendsen H. J. C. GROMACS: Fast, Flexible, and Free. J. Comput. Chem. 2005, 26, 1701–1718. 10.1002/JCC.20291. 16211538

[ref76] Ivani I.; Dans P. D.; Noy A.; Pérez A.; Faustino I.; Hospital A.; Walther J.; Andrio P.; Goñi R.; Balaceanu A.; Portella G.; Battistini F.; Gelpí J. L.; González C.; Vendruscolo M.; Laughton C. A.; Harris S. A.; Case D. A.; Orozco M. Parmbsc1: A Refined Force Field for DNA Simulations. Nat. Methods 2016, 13, 55–58. 10.1038/NMETH.3658. 26569599 PMC4700514

[ref77] Eswar N.; Webb B.; Marti-Renom M. A.; Madhusudhan M. S.; Eramian D.; Shen M.; Pieper U.; Sali A. Comparative Protein Structure Modeling Using MODELLER. Curr. Protoc. Protein Sci. 2007, 50, 2.9.1–2.9.31. 10.1002/0471140864.PS0209S50. 18429317

[ref78] Rodrigues J.; Valentine C.; Jimenez B. JoaoRodrigues/Interfacea: First Beta Version of the API; Zenodo, 2019 10.5281/ZENODO.3516439.

[ref79] The MathWorks Inc. MATLAB, version: 9.13.0 (R2022b); MathWorks Inc.: Natick, Massachusetts, 2022.

[ref80] Angelov D.; Lenouvel F.; Hans F.; Muller C. W.; Bouvet P.; Bednar J.; Moudrianakis E. N.; Cadet J. L.; Dimitrov S. The Histone Octamer Is Invisible When NF-KAPPAB Binds to the Nucleosome. Biochem. Mol. Biol. 2004, 279, 42374 10.1074/jbc.M407235200. 15269206

