# Supplementary material for: Molecular Mechanism of Nucleosome Recognition by the Pioneer Transcription Factor Sox
Source: J Chem Inf Model. 2023 Jun 12;63(12):3839–53. doi: 10.1021/acs.jcim.2c01520 (PMC10302475; doi:10.1021/acs.jcim.2c01520)
Supplement: Supplementary file 1 — ci2c01520_si_001.pdf [file ci2c01520_si_001.pdf]

SUPPORTING INFORMATION

Molecular Mechanism of Nucleosome Recognition by the Pioneer Transcription Factor Sox

Burcu Ozden[1,2+], Ramachandran Boopathi[3,4,5+], Ayşe Berçin Barlas[1,2], Imtiaz N. Lone[2], Jan Bednar[3], Carlo Petosa[4], Seyit Kale[1], Ali Hamiche[6*], Dimitar Angelov[1,5*], Stefan Dimitrov[1,3,7*], Ezgi Karaca[1,2*]

[1]Izmir Biomedicine and Genome Center, Dokuz Eylul University Health Campus, Izmir 35340, Turkey
[2]Izmir International Biomedicine and Genome Institute, Dokuz Eylül University, Izmir, 35340, Turkey
[3]Institut for Advanced Biosciences, Inserm U 1209, CNRS UMR 5309, Université Grenoble Alpes, Grenoble 38000, France
[4]Institut de Biologie Structurale (IBS), Université Grenoble Alpes, CEA, CNRS, Grenoble 38044, France
[5]Université de Lyon, Ecole Normale Supérieure de Lyon, CNRS, Laboratoire de Biologie et de Mode´ lisation de la Cellule LBMC, 46 Allée d'Italie, Lyon, 69007, France
[6]Département de Génomique Fonctionnelle et Cancer, Institut de Génétique et Biologie Moléculaire et Cellulaire (IGBMC)/Université de Strasbourg/CNRS/INSERM, Illkirch Cedex, 67404, France.
[7] Roumen Tsanev Institute of Molecular Biology, Bulgarian Academy of Sciences, Sofia, Bulgaria

[+]These authors have contributed equally to this work.

[*] Corresponding authors: Ezgi Karaca, Stefan Dimitrov, Ali Hamiche, Dimitar Angelov

Correspondence: Ezgi Karaca. Tel:+905334566096; Fax:+90 02322776353; Email: ezgi.karaca@ibg.edu.tr

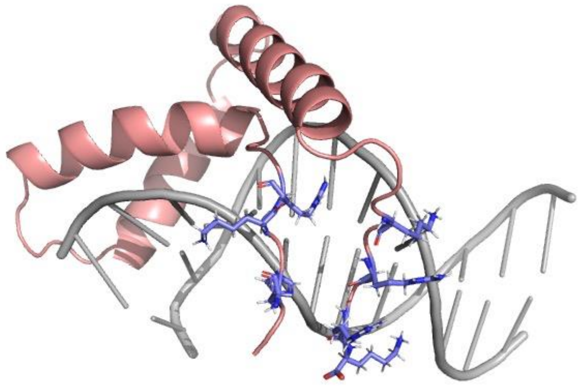

46--SGHIKRPMNAF-BoomerangShape-YPDYKYRPRK--122

Figure S1. The basic residues of Sox tails contribute to stable binding of Sox to its cognate DNA. The protein sequence spans the amino acid range from 46 and 122 of Sox11. The basic tail residues are highlighted in violet. The folded boomerang-shaped a-helical region is represented as a box (PDB ID: 6t78).

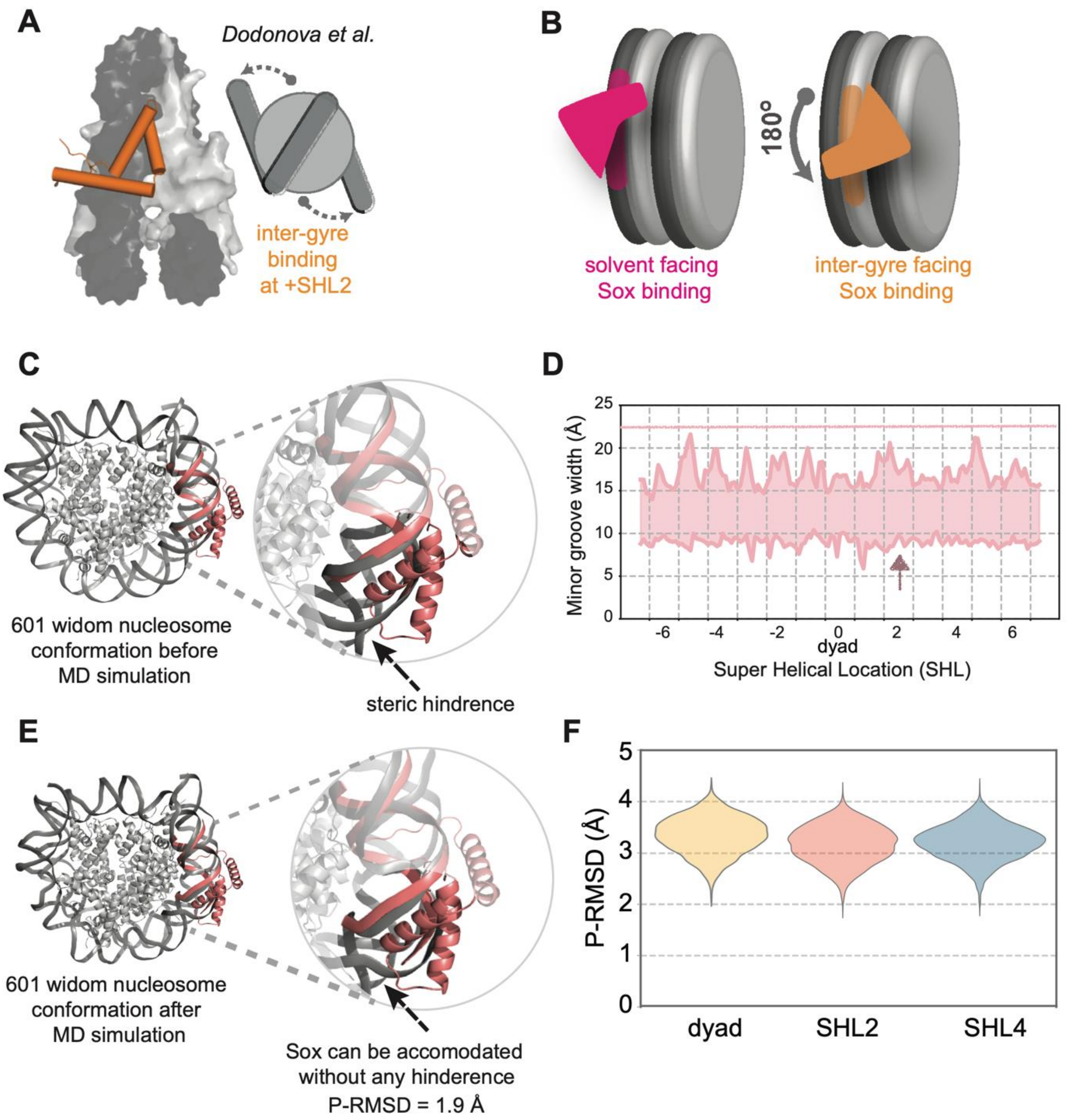

Figure S2. A. Dodonova *et al.*'s Sox:NCP binding mode (pdb id: 6t7b). Sox binding site is located at the inter-gyre facing strand at SHL2. Sox is shown in orange. **B.** The orientation of Sox differs according to the placement of its recognition sequence. If the Sox binding sequence is placed on the solvent facing DNA strand (left), Sox should bind to the nucleosome without imposing major structural alterations. **C.** Sox:DNA complex is structurally aligned onto the Sox recognition sequence, inserted at SHL2. The resulting fitting is highlighted, showing that when free and nucleosomal DNA conformations are matched, the SHL2 minor groove is too narrow to accommodate Sox (pink). **D.** The minor groove width profile variations of the available nucleosome structures. The signature Sox binding signal requires a peak at 22.5 Å minor groove widening, noted with a pink solid line. Such widening could not be observed at SHL2, marked with an arrow (The shaded area reflects the minor groove width fluctuation ranges, sampled by 1aoi, 1eqz, 1f66, 1id3, 1kx3, 1kx4, 1kx5, 1m18, 1m19, 1m1a, 1p34, 1p3a, 1p3b, 1p3f, 1p3g, 1p3i, 1p3k, 1p3l, 1p3m, 1p3o, 1p3p, 1s32, 1u35, 1zla, 2cv5, 2fj7, 2nqb, 2nzd,

2pyo, 3a6n, 3afa, 3an2, 3av1, 3av2, 3ayw, 3aze, 3azf, 3azg, 3azh, 3azi, 3azj, 3azk, 3azl, 3azm, 3azn, 3b6f, 3b6g, 3c1b, 3kuy, 3kwq, 3kxb, 3lel, 3lja, 3lz0, 3lz1, 3mgp, 3mgq, 3mgr, 3mgs, 3mnn, 3mvd, 3o62, 3reh, 3rei, 3rej, 3rek, 3rel, 3tu4, 3ut9, 3uta, 3utb, 3w96, 3w97, 3w98, 3w99, 3wa9, 3waa, 3wkj, 3wtp, 3x1s, 3x1t, 3x1u, 3x1v, 4jjn, 4kgc, 4kud, 4ld9, 4qlc, 4r8p, 4wu8, 4wu9, 4x23, 4xuj, 4xzq, 4ym5, 4ym6, 4ys3, 4z5t, 4z66, 4zux, 5av5, 5av6, 5av8, 5av9, 5avb, 5avc, 5ay8, 5b0y, 5b0z, 5b1l, 5b1m, 5b24, 5b2i, 5b2j, 5b31, 5b32, 5b33, 5b40, 5cp6, 5cpi, 5cpj, 5cpk, 5dnm, 5dnn, 5e5a, 5f99, 5gse, 5gsu, 5gt0, 5gt3, 5gtc, 5gxq, 5hq2, 5jrg, 5kgf, 5mlu, 5nl0, 5o9g, 5omx, 5ong, 5onw, 5wcu, 5x0x, 5x0y, 5x7x, 5xf3, 5xf4, 5xf5, 5xf6, 5xm0, 5xm1, 5y0c, 5y0d, 5z23, 5z30, 5z3l, 5z3t, 5z3u, 5z3v, 5zbx, 6a5l, 6a5o, 6a5p, 6a5r, 6a5t, 6a5u, 6buz, 6c0w, 6dzt, 6e0c, 6e0p, 6esf, 6esg, 6esh, 6esi, 6fml, 6fq5, 6fq6, 6fq8, 6gej, 6gen, 6hkt, 6hts, 6i84, 6inq, 6ir9, 6iro, 6iy3, 6j4w, 6j4x, 6j4y, 6j4z, 6j50, 6j51, 6j99, 6jm9, 6jma, 6jyl, 6k1p, 6muo, 6mup, 6ne3, 6nj9, 6nn6, 6nog, 6nqa, 6o1d, 6o96, 6om3, 6r1t, 6r1u, 6r25, 6r8y, 6r8z, 6r90, 6r91, 6r92, 6r93, 6r94). **E.** After simulating the mutant 601 Widom nucleosome, SHL2 could accommodate Sox (pink) without any clashes. **(F)** The Phosphate Root Mean Square Deviation (P-RMSD) distributions of 601-SHL024 throughout the simulation (N=3000). P-RMSD values are calculated by taking the Sox11-DNA conformation as a reference (pdb id: 6t78). The lowest P-RMSD values are 2.1 Å, 1.9Å and 1.9Å for dyad, SHL2, and SHL4, respectively.

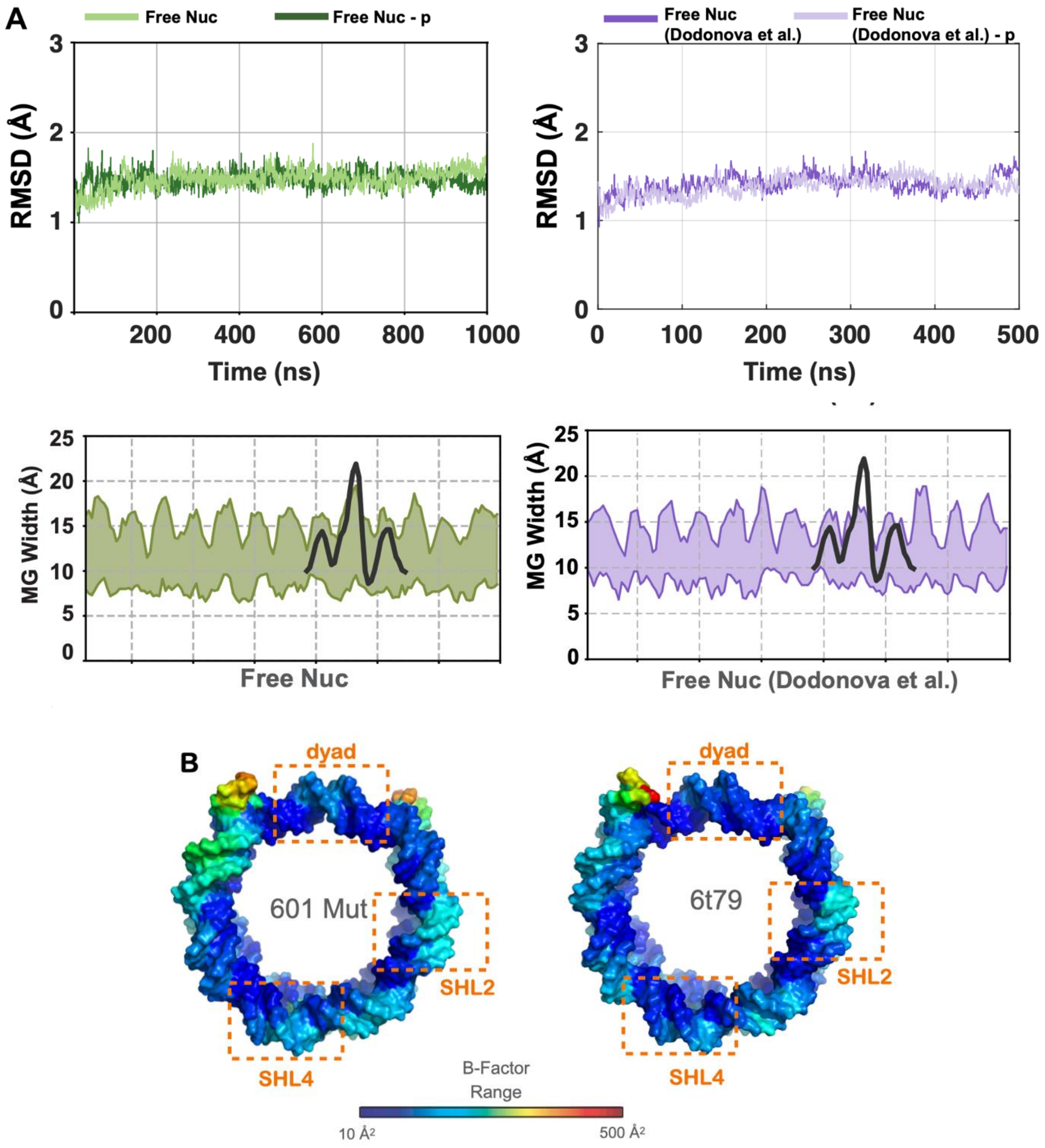

Figure S3. A. The MD-driven RMSD and minor groove (MG) width fluctuations of free 601 mutated and free NCAP-SELEX nucleosome of Dodonova *et al.* (pdb id: 6t79). Each replica simulation is explicitly represented in the RMSD profiles (p denotes parallel), while in the MG profiles, they are pooled together. The reference Sox-bound MG width profile (calculated on 6t79) is demonstrated with a dark tick line. **B.** RMSF-driven B-factor profiles are mapped on the mutated 601 and 6t79 nucleosomal DNA.

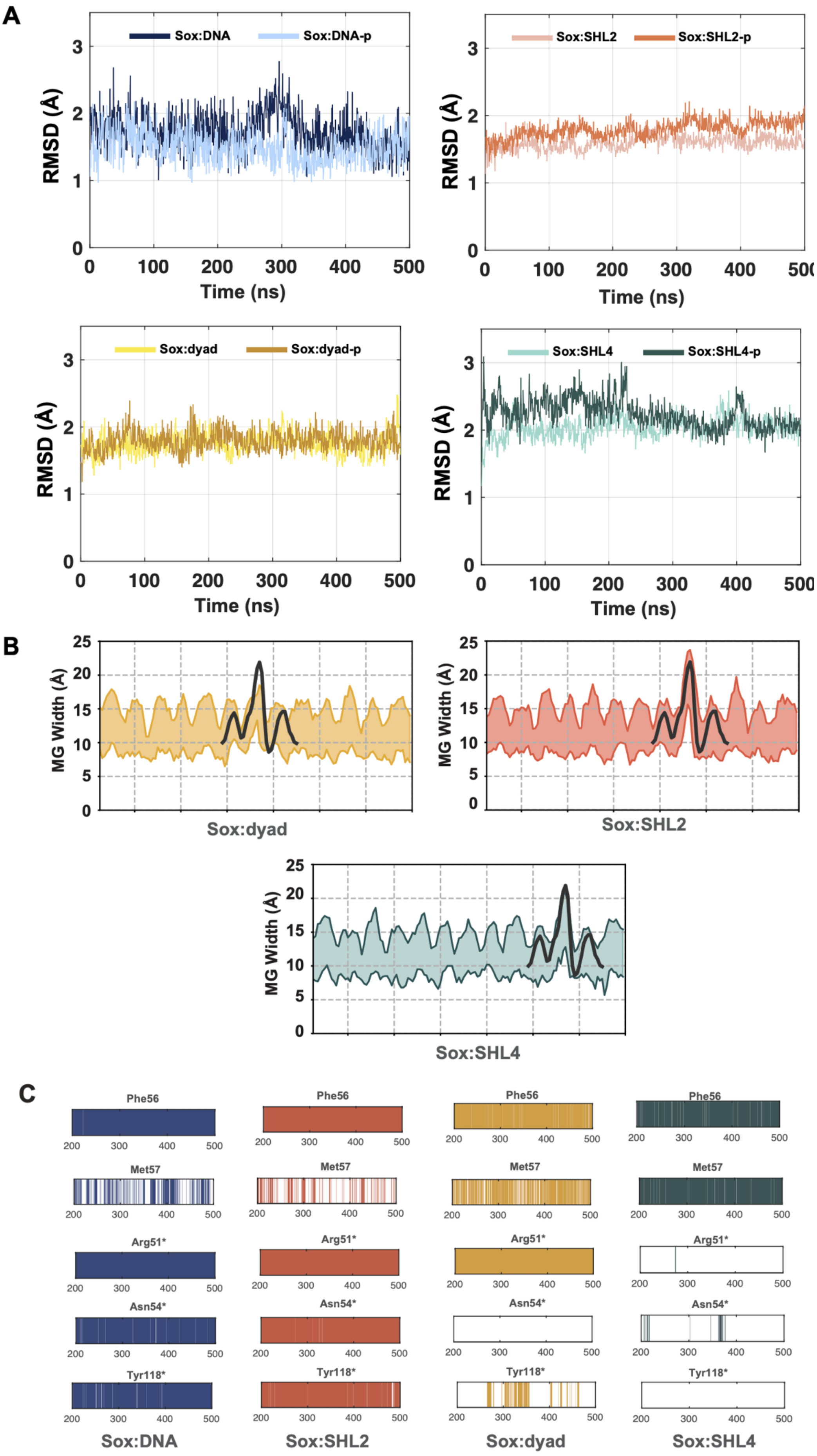
A
Sox:DNA
Sox:DNA-p
Sox:SHL2
Sox:SHL2-p
Sox:dyad
Sox:dyad-p
Sox:SHL4
Sox:SHL4-p
RMSD (Å)
Time (ns)
B
MG Width (Å)
Sox:dyad
Sox:SHL2
Sox:SHL4
C
Phe56
Met57
Arg51*
Asn54*
Tyr118*
Sox:DNA
Sox:SHL2
Sox:dyad
Sox:SHL4

Figure S4. A. The MD-driven RMSD profiles of Sox:free DNA (blue), Sox:SHL2 (red), Sox:dyad (yellow), Sox:SHL4 (green). Each replica simulation is explicitly represented in the RMSD profiles (p denotes parallel). **B.** The minor groove (MG) width fluctuations of Sox:free DNA (blue), Sox:SHL2 (red), Sox:dyad (yellow), Sox:SHL4 (green). The replica simulations are pooled together. The reference Sox-bound MG width profile is demonstrated with a dark tick line. **C.** The reference profile of Sox SHL2 bound nucleosome structure (pdb id: 6t7b) is shown in dark gray and placed at the relevant SHLs to serve as a comparison. **C.** The Sox11:DNA interaction profiles of the essential Sox amino acids observed in our replica simulations, at free DNA, SHL2, dyad, and SHL4 sites (N=600 for each complex). The percentages of these interactions are given in Table S1.

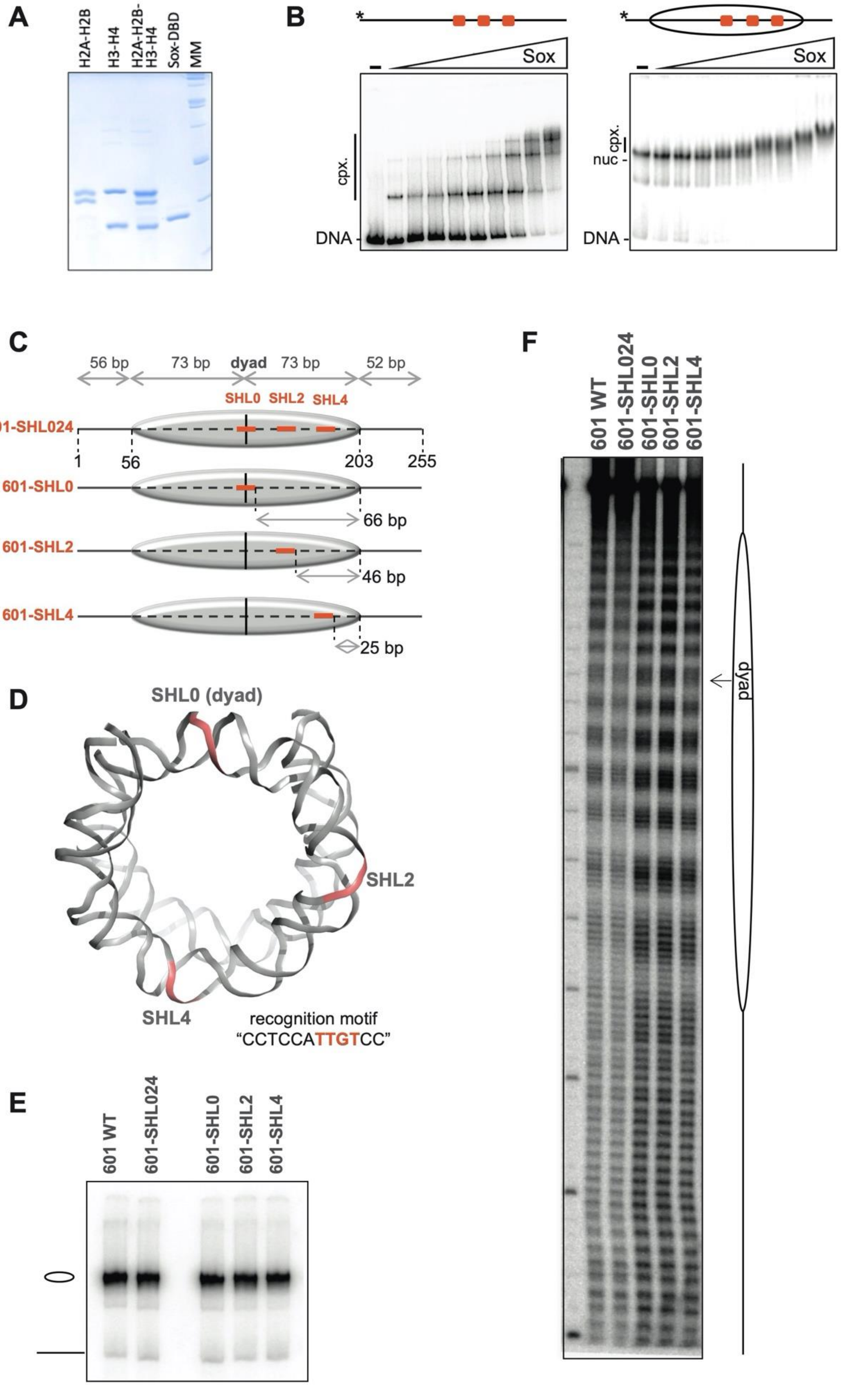

Figure S5. A. DNA Electrophoretic analysis of the purified recombinant histones, the reconstituted histone octamer and the purified Sox6 HMG-domain. **B.** EMSA showing the binding of Sox6 HMG-domain to naked DNA (601-SHL024) (left) or to nucleosome (601-SHL024) (right). Naked ^{32}P-end

labeled 601-SHL024 DNA or nucleosomes were incubated with increasing amount of Sox6 HMG-domain and aliquots of the reaction mixtures were run on a native PAGE. For each experimental condition, the concentration of the Sox6 HMG-domain used in the nucleosome binding experiments was ~ 3-fold higher compared to the one used for analyzing the binding to naked DNA. The positions of free DNA, nucleosomes and their complexes with Sox6 HMG-domain are indicated. **C.** Schematics of the reconstituted nucleosomes. The Sox binding site was inserted in the 255 bp 601 DNA fragment at three different locations, namely at the dyad (SHL0), at SHL2 and at SHL4. Bold lines correspond to free DNA arms; oval to core particle region; vertical black line to nucleosome dyad; salmon bold line to Sox binding sites. The numbers refer to the length of DNA in the depicted regions. **D.** 601 nucleosomal DNA sequence (pdb id: 3lz0) is mutated upon inserting Sox binding sequence, 5'-*CCTCCA**TTGT**CC-3',* at three different SHLs, namely dyad, SHL2 and SHL4. All the nucleosome inserted Sox binding sites face the solution. The three Sox recognition DNA sequences are colored in salmon. **E.** EMSA of the indicated reconstituted nucleosomes. The positions of free DNA (horizontal black line) and reconstituted nucleosomes (oval) are indicated. **F.** Hydroxyl radical footprinting of the reconstituted nucleosomes. First line, molecular mass marker; Schematics of the nucleosome is shown on the right. The dyad is indicated with an arrow.

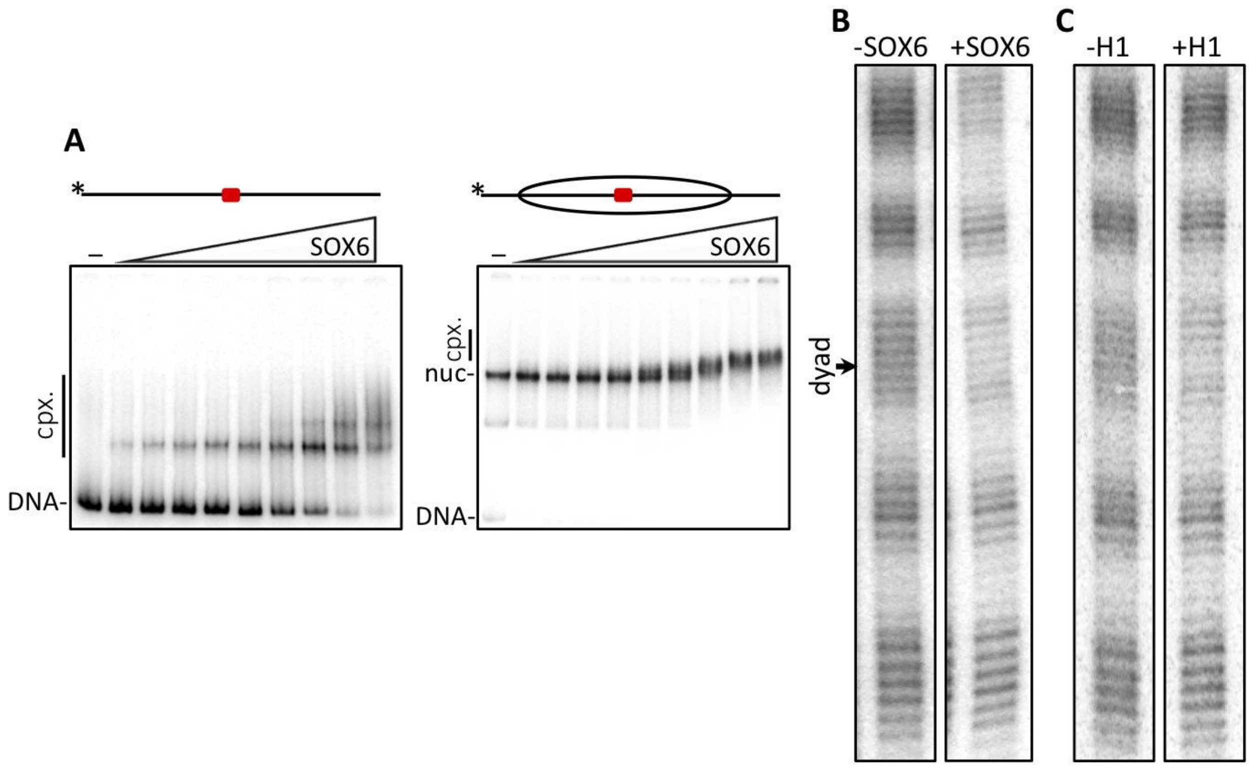

Figure S6. A. EMSA showing the binding of Sox6 HMG-domain to naked naked (601-SHL0) DNA (left) and (601-SHL0) nucleosome (right). Naked 32P-end labeled 601-SHL024 DNA or nucleosomes were incubated with increasing amount of Sox6 HMG-domain and aliquots of the reaction mixtures were run on a native PAGE. For each experimental condition, the concentration of the Sox6 HMG-domain used in the nucleosome binding experiments was ~ 3-fold higher compared to the one used for analyzing the binding to naked DNA. The positions of free DNA, nucleosomes and their complexes with Sox6 HMG-domain are indicated. **B.** •OH radical DNA cleavage pattern of the region around the dyad of 601 nucleosome with bound (+) and unbound (-) linker Sox6. A clear footprint in the Sox6 bound nucleosome is observed at the dyad. **C.** •OH radical DNA cleavage pattern of the region around the dyad of the 601-SHL024 nucleosome in the absence (-) or in the presence (+) bound H1. The dyad is indicated by an arrow. Note the clear footprint around the dyad of the H1-bound nucleosome. The data shows a very similar footprint of Sox and H1 at the dyad of the nucleosome.

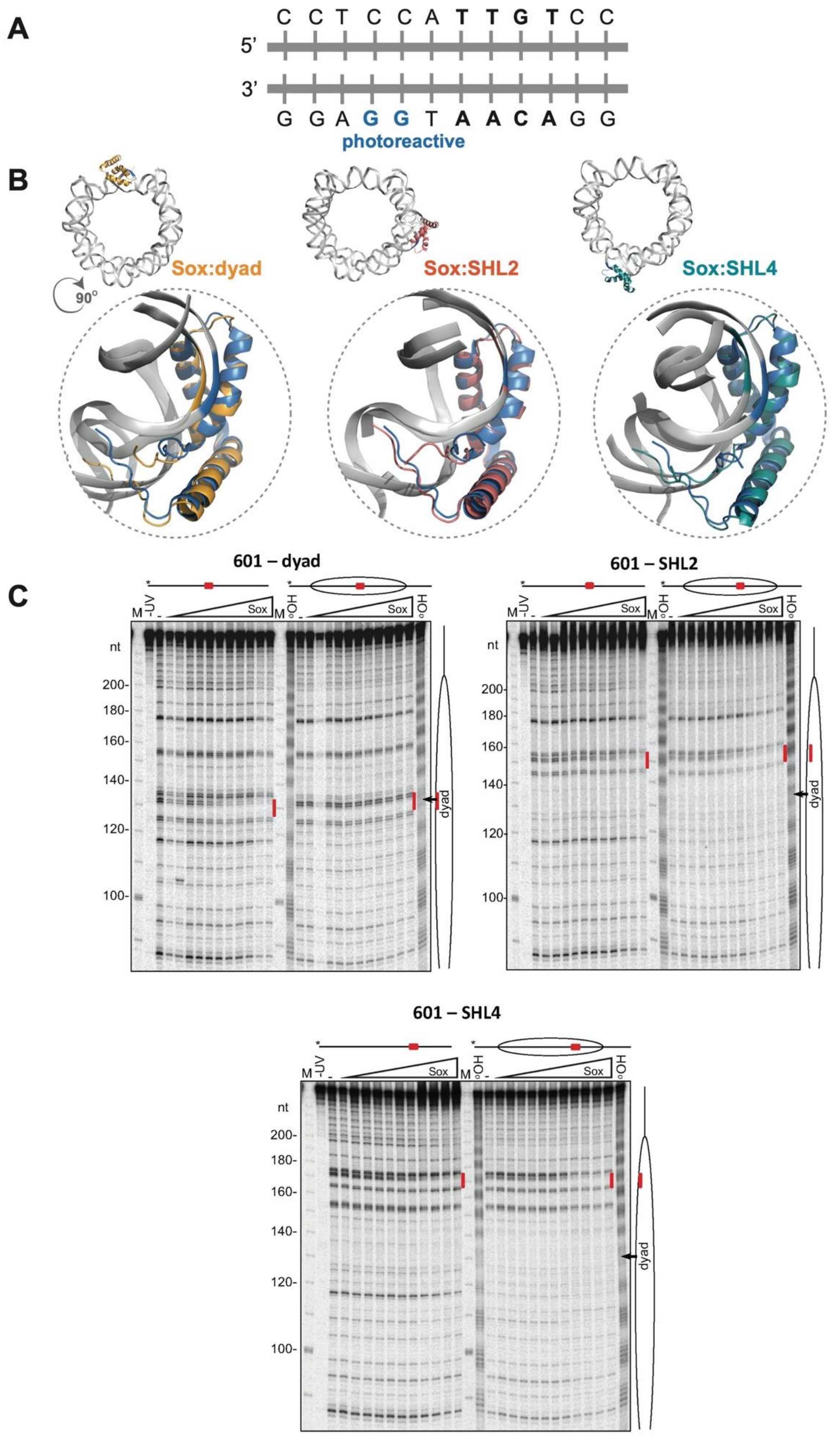

Figure S7. A. The representative coordinates of Sox cognate binding sequence and photoreactive GG nucleotides. Sox cognate sequence "TTGT" is shown as bold and photoreactive GG nucleotides are depicted in blue. **B.** The representative location of photoreactive GG nucleotides on SHLs. GG

deformation of Sox:nucleosome models are compared with Sox:DNA (pdb id:4y60). Reference is depicted with blue color and Sox:nucleosome at dyad, SHL2 and SHL4 models are presented in orange, pink and green, respectively. **C.** UV laser footprinting patterns of the Sox-DNA and Sox-nucleosome complexes bearing a Sox recognition sequence either at the dyad (left), at SHL2 (right), and at SHL4 (below-middle), respectively. The complexes were irradiated with a single 5 nanoseconds UV laser 266 nm pulse (Epulse,0.1 J/cm^2) and DNA was purified from the samples. After treatment of the purified DNA with Fpg glycosylase, the cleaved DNA fragments were separated on 8% sequencing gel and visualized by autoradiography. Red vertical lines and red squares mark the Sox binding sites, M marks the molecular mass, oval represent the schematics of the nucleosome, and the dyad is indicated with an arrow. -UV refers to the control, non-UV irradiated and Fpg treated sample.

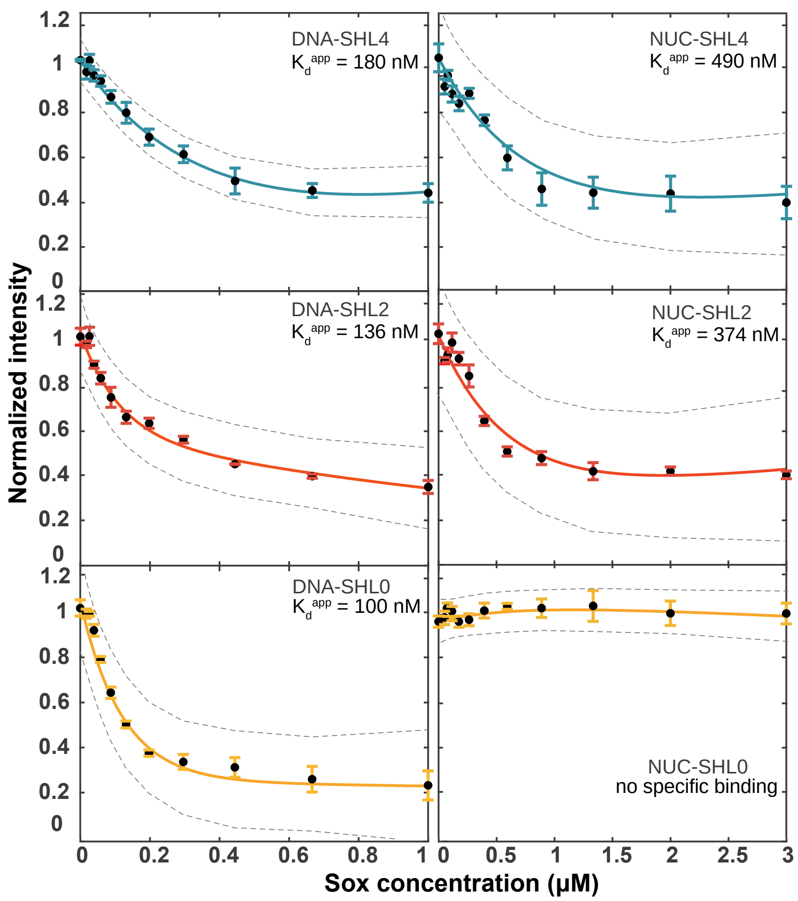

Figure S8. Sox6 concentration dependences of the footprinting intensity representing the normalized cleavage band intensity of the GG run within the binding site for DNA (left) and Nucleosome (right) bound Sox at SHL4, SHL2 and SHL0 individually. The curve fitting equation follows f1(x) = a*exp(b*x) + c*exp(d*x) function, with the following parameters:

**Parameter**	**DNA-SHL024-SHL0**	**DNA-SHL024-SHL2**	**DNA-SHL024-SHL4**	**NUC-SHL024-SHL0**	**NUC-SHL024-SHL2**	**NUC-SHL024-SHL4**
**a**	1.050	1.024	1.016	0.222	1.037	1.069
**b**	-0.0027	-0.0015	-0.0023	-6.4594e-4	-8.163e-4	-6.697e-4
**c**	0.0018	1.94e-8	0.0340	0.7894	0.0389	0.0031
**d**	0.0048	0.0156	0.0018	4.335e-5	7.085e-4	0.0014
**R^2**	0.980	0.976	0.958	0.673	0.959	0.955
**RMSE**	0.051	0.045	0.067	0.027	0.0634	0.069

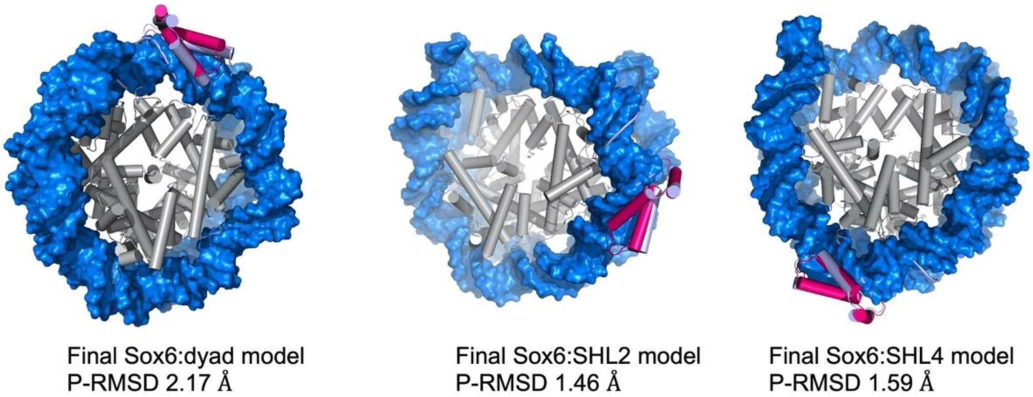

Figure S9. The lowest P-RMSD Sox6:NCP complexes. The reference Sox:DNA complex (pdb id:6T78) is depicted in lilac.

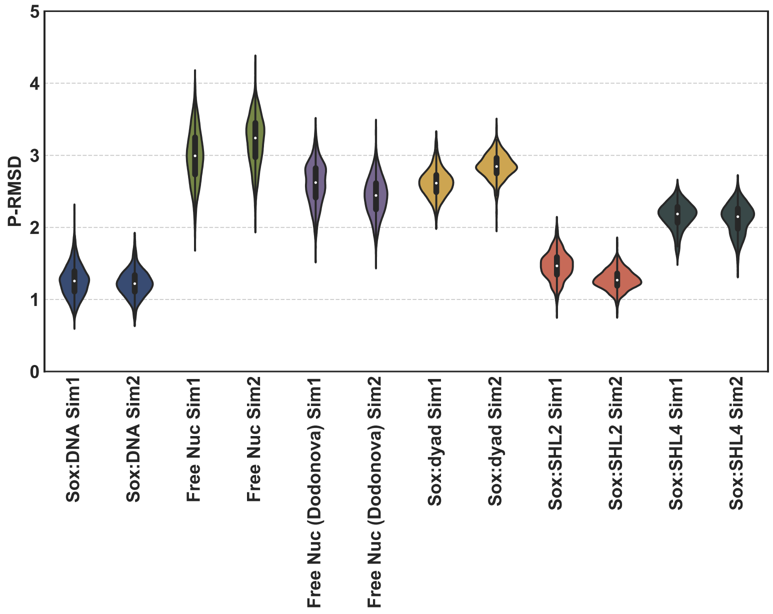

Figure S10. Individual depiction of minor groove P-RMSD evaluations of free and Sox11-bound simulations.

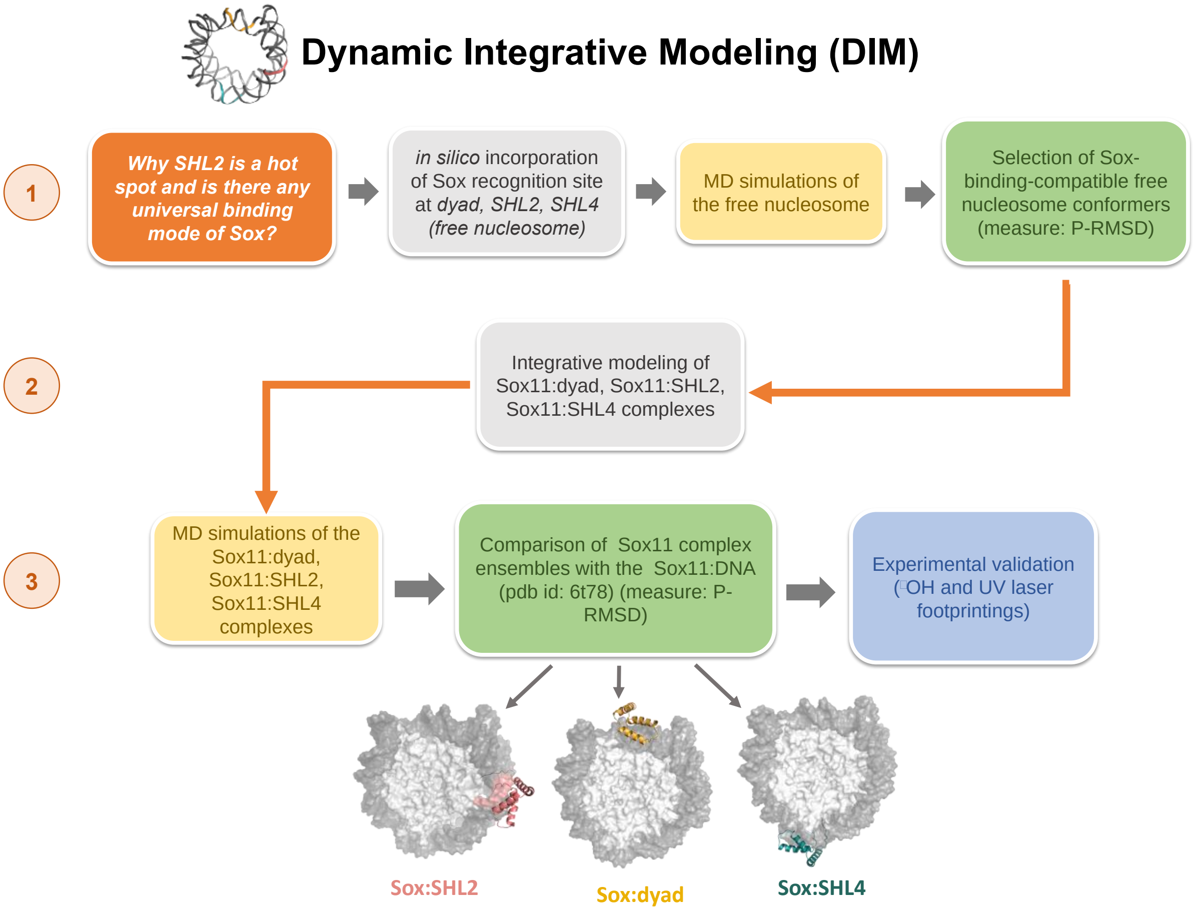

Figure S11. Dynamic Integrative Modeling (DIM) pipeline. The steps followed during our DIM protocol are indicated within boxes. The orange box is our starting point; yellow and gray boxes refer to MD simulations and modeling steps, respectively; green boxes depict the checkpoints, where we chose a representative structure from the generated conformation pool and blue box represents the experimental validation of our results using ˙OH and UV laser footprintings techniques.

Table S1. Sox11:DNA interaction percentages of the essential Sox amino acids. For each complex, the percentages of each replica simulation and their average value are provided. Hydrophobic and base specific h-bond interactions are shown in green and purple, respectively. * represents base specific h-bond interactions. The generic (non-specific) interactions are shown in black.

Sox11 amino acids	DNA:Sox			Sox:SHL2			Sox:dyad			Sox:SHL4		
	Sim1	Sim2	Avg.	Sim1	Sim2	Avg.	Sim1	Sim2	Avg.	Sim1	Sim2	Avg.
**Phe56**	**100.0**	**99.8**	**99.9**	**100.0**	**100.0**	**100**	**96.8**	**96.3**	**96.55**	**92.0**	**95.8**	**93.9**
**Met57**	**54.2**	**39.3**	**46.75**	**42.1**	**25.3**	**33.7**	**68.9**	**74.0**	**71.45**	**87.7**	**97.2**	**92.45**
Arg51	100.0	100.0	100	100.0	100.0	100	99.7	100.0	99.85	85.5	96.3	90.9
**Arg51***	**100.0**	**100.0**	**100**	**100.0**	**100.0**	**100**	**70.0**	**100.0**	**85**	**77.2**	**0.5**	**38.85**
Asn54	100.0	100.0	100	100.0	100.0	100	30.3	43.6	36.95	5.8	24.8	15.3
**Asn54***	**99.2**	**97.8**	**98.5**	**100.0**	**99.3**	**99.65**	**0.2**	**0.0**	**0.1**	**0.0**	**4.8**	**2.4**
Tyr118	99.8	100.0	99.9	99.5	99.7	99.6	91.7	69.6	80.65	17.5	28.1	22.8
**Tyr118***	**98.3**	**97.0**	**97.65**	**98.0**	**96.8**	**97.4**	**43.3**	**21.6**	**32.45**	**2.2**	**0.0**	**1.1**

Table S2. The Sox11 amino acids that fall within 7Å of the nearest histone protein. The calculations are made on the complexes, fitting to the Sox:DNA binding conformation the best.

Sox11:SHL2	Sox11:dyad	Sox11:SHL4
ARG121 (C-tail)<br>LYS122 (C-tail)	SER46 (globular)<br>LYS81 (globular)<br>LYS88 (globular)<br>ARG121 (C-tail)<br>LYS122 (C-tail)	HIS75(globular)<br>ASN76 (globular)<br>ALA77 (globular)<br>GLU78 (globular)<br>LYS81 (globular)<br>LYS85 (globular)<br>TYR118 (C-tail)<br>PRO120 (C-tail)<br>ARG121 (C-tail)<br>LYS122 (C-tail)

Table S3. The complete table of simulated nucleosome and Sox complexes.

System	Simulation No	Simulation Time (ns)
Free mutated 601 nucleosome	Simulation 1	1,000
	Simulation 2	1,000
Free NCAP-SELEX nucleosome (Dodonova *et al.*, pdb id:6t79)	Simulation 1	500
	Simulation 2	500
Sox:freeDNA (pdb id: 6t78)	Simulation 1	500
	Simulation 2	500
Sox11:SHL2	Simulation 1	500
	Simulation 2	500
Sox11:dyad	Simulation 1	500
	Simulation 2	500
Sox11:SHL4	Simulation 1	500
	Simulation 2	500
Sox6:SHL2	Simulation 1	500
Sox6:dyad	Simulation 1	500
Sox6:SHL4	Simulation 1	500

SUPPORTING MOVIES:

The link to the supporting movies is https://github.com/CSB-KaracaLab/Sox-PTF/tree/main/Movies

SHL2.mov corresponds to the binding mechanism of Sox11 at SHL2; SHL4.mov to Sox at SHL4 and dyad.mov to Sox at dyad, as outlined by our dynamic integrative modeling. The green residues indicate the essential polar Sox amino acids, while the purple ones refer to the hydrophobic ones.
